# Tuning the local chemical environment of ZnSe quantum dots with dithiols towards photocatalytic CO_2_ reduction†

**DOI:** 10.1039/d2sc00890d

**Published:** 2022-04-11

**Authors:** Constantin D. Sahm, Anna Ciotti, Eric Mates-Torres, Vivek Badiani, Kamil Sokołowski, Gaia Neri, Alexander J. Cowan, Max García-Melchor, Erwin Reisner

**Affiliations:** Yusuf Hamied Department of Chemistry, University of Cambridge Lensfield Rd Cambridge CB2 1EW UK reisner@ch.cam.ac.uk; School of Chemistry, CRANN and AMBER Research Centres, Trinity College Dublin, College Green Dublin 2 Ireland; Stephenson Institute for Renewable Energy, Department of Chemistry, The University of Liverpool Crown Street Liverpool L69 7ZD UK garciamm@tcd.ie

## Abstract

Sunlight-driven CO_2_ reduction to renewable fuels is a promising strategy towards a closed carbon cycle in a circular economy. For that purpose, colloidal quantum dots (QDs) have emerged as a versatile light absorber platform that offers many possibilities for surface modification strategies. Considerable attention has been focused on tailoring the local chemical environment of the catalytic site for CO_2_ reduction with chemical functionalities ranging from amino acids to amines, imidazolium, pyridines, and others. Here we show that dithiols, a class of organic compounds previously unexplored in the context of CO_2_ reduction, can enhance photocatalytic CO_2_ reduction on ZnSe QDs. A short dithiol (1,2-ethanedithiol) activates the QD surface for CO_2_ reduction accompanied by a suppression of the competing H_2_ evolution reaction. In contrast, in the presence of an immobilized Ni(cyclam) co-catalyst, a longer dithiol (1,6-hexanedithiol) accelerates CO_2_ reduction. ^1^H-NMR spectroscopy studies of the dithiol-QD surface interactions reveal a strong affinity of the dithiols for the QD surface accompanied by a solvation sphere governed by hydrophobic interactions. Control experiments with a series of dithiol analogues (monothiol, mercaptoalcohol) render the hydrophobic chemical environment unlikely as the sole contribution of the enhancement of CO_2_ reduction. Density functional theory (DFT) calculations provide a framework to rationalize the observed dithiol length dependent activity through the analysis of the non-covalent interactions between the dangling thiol moiety and the CO_2_ reduction intermediates at the catalytic site. This work therefore introduces dithiol capping ligands as a straightforward means to enhance CO_2_ reduction catalysis on both bare and co-catalyst modified QDs by engineering the particle's chemical environment.

## Abstract 2

ZnSe quantum dots (yellow sphere) are modified with dithiols of various lengths for enhanced visible light-driven CO_2_ to CO reduction in either the absence or presence of a molecular Ni co-catalyst.

## Graphical abstract

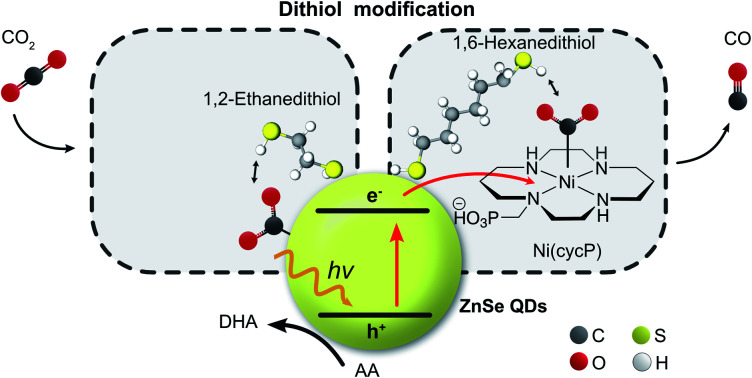

## Introduction

Converting CO_2_ into renewable fuels driven by solar light can contribute to alleviating the global dependence on fossil fuels.^1,2^ Colloidal quantum dots (QDs) have emerged during the last decade as light absorbers for the photocatalytic H_2_ evolution reaction (HER)^3^ and CO_2_ reduction reaction (CO_2_RR).^4^ Molecular co-catalysts based on transition metal complexes are often employed in combination with colloidal QDs to facilitate the kinetically challenging multi-electron CO_2_RR,^5–7^ but approaches without an additional co-catalyst are also known and include hetero-atom doping^8^ or surface modification strategies.^9,10^ ZnSe QDs are thereby well suited because their direct band gap of 2.7 eV enables absorption of near-UV and visible light while the conduction band is located at −1.4 V (*vs.* NHE at pH 5.5),^11^ which is sufficiently reductive to enable CO_2_ photoreduction using molecular co-catalysts or the QD surface itself.

Approaches that go beyond the intrinsic optimization of the catalytic site and expand into the secondary coordination sphere to stabilize reaction intermediates are increasingly governing the design of CO_2_ reduction electro- and photocatalysts.^12,13^ A plethora of chemical functionalities have been reported to influence the interfacial CO_2_RR stretching from amino acids^14^ to imidazolium groups,^15–17^ amines,^18,19^ pyridines,^20^ as well as *N*-heterocyclic carbenes^21^ and *N*-arylpyridinium salts.^22^ Furthermore, capping ligands with a dangling alkyl chain were employed as surface modifiers to introduce a hydrophobic environment to trap CO_2_ and allow a higher substrate concentration at the catalytic site.^23^ Thiols are a commonly used anchoring group amongst capping ligands due to the strong affinity of the thiol to soft metal surface sites and can be used to introduce a dangling chemical functionality in proximity to the colloidal nanocrystals.^24^ Dithiols feature two thiol groups and have been explored as nanocrystal capping, multidentate anchors with a stronger affinity compared to monodentate thiols,^25,26^ cross-linking agents,^27^ and as hole quenchers,^28^ but they remain unexplored in the field of CO_2_RR.

**Fig. 1:**
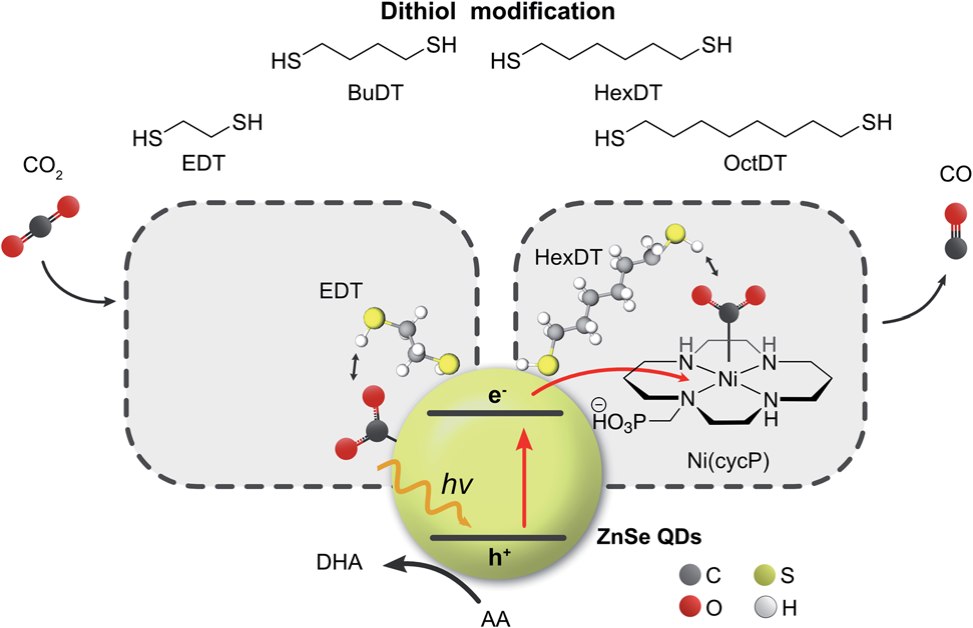
Schematic representation of the photocatalyst system consisting of ligand-free ZnSe–BF_4_ QDs (yellow sphere, BF_4_^−^ omitted for clarity) modified with dithiols of various lengths for visible light-driven CO_2_ to CO reduction in either the absence or presence of a molecular co-catalyst Ni(cycP). Ascorbic acid (AA) is used as the sacrificial electron donor. DHA: dehydroascorbic acid.

Inspired by our previous work^7,17^ that the surface of ZnSe QDs can be tailored towards the CO_2_RR by surface-modification with an imidazolium moiety, we herein show that dithiols can influence interfacial CO_2_ photoreduction facilitated by ZnSe QDs (Fig. 1). The dithiol-QD interactions are examined systematically by ^1^H-NMR spectroscopy and dynamic light scattering, which reveal a solvation sphere dominated by hydrophobic interactions involving the dithiols. Under photoreduction conditions, the presence of short dithiols promotes CO_2_RR on the unfunctionalized ZnSe QDs while the presence of a long dithiol improves CO_2_RR when a molecular co-catalyst is used as the main catalytic site. A systematic survey of dithiols and mercaptoalcohol/monothiol analogues shows that the second thiol moiety is essential for the observed effects. Finally, DFT calculations shed light on the length-dependent activity enhancement of the dithiols in the presence and absence of the molecular co-catalyst.

## Results and discussion

### Synthesis and characterization of ZnSe QDs

ZnSe QDs were prepared as reported previously^7^ by heating zinc stearate and selenium in octadecene to 300 °C followed by reactive ligand stripping^29^ using Me_3_OBF_4_ to replace stearate by weakly coordinating BF_4_^−^ anions (ZnSe–BF_4_, see Fig. S1† and ESI† for full characterization) from the surface. Transmission electron microscopy (TEM) shows highly crystalline, pseudo-spherical particles with a diameter of 4.5 ± 0.7 nm. The particles feature a strong visible-light response with a first excitonic absorption maximum at 416 nm, determined by UV-vis spectroscopy. Powder X-ray diffraction confirms a zinc blende crystal structure accompanied by broadening due to nanostructuring.

### Photocatalytic CO_2_ reduction

**Fig. 2:**
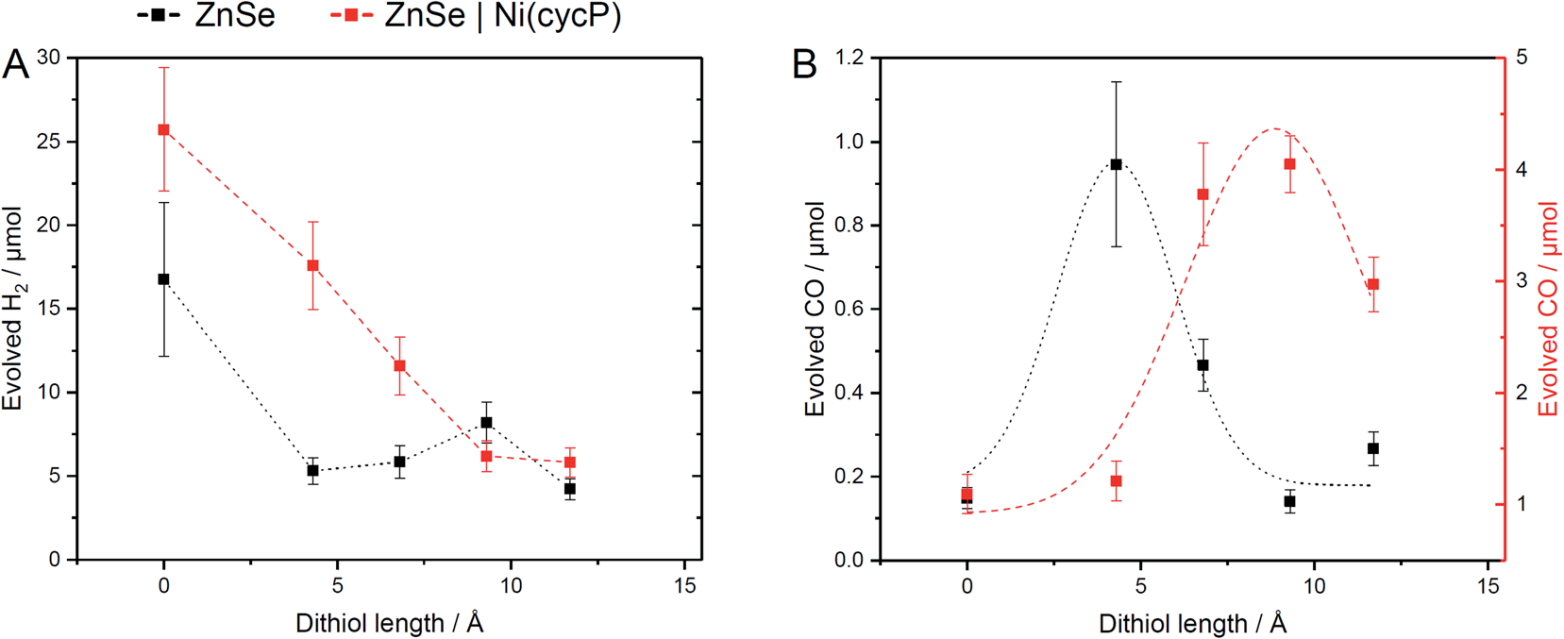
Influence of dithiol ligands on photocatalytic CO_2_RR using ZnSe–BF_4_ QDs. The details of the structure and length of the employed dithiols are provided in Table 1 and Fig. 1. (A) Evolved H_2_ and (B) evolved CO. Conditions: ZnSe|dithiol: 50 μM dithiol, pH 6.5; ZnSe|Ni(cycP)|dithiol: 25 μM dithiol, 10 μM Ni(cycP), pH 5.5. General conditions: AM 1.5 G, *λ* > 400 nm, 100 mW cm^−2^, 10 h irradiation, 0.5 μM ZnSe–BF_4_, 0.1 M AA/NaHCO_3_, CO_2_ constant flow (4 sccm), 25 °C. The dashed lines serve to guide the eye. The full photocatalysis dataset can be found in Fig. S2 and S3.†

The influence of dithiols on photocatalytic CO_2_ to CO reduction was systematically studied with two well-established systems based on ZnSe–BF_4_ QDs.^7,17^ More specifically, photocatalytic CO_2_RR was investigated on a bare ZnSe surface with and without the presence of an immobilized molecular co-catalyst, *i.e.* phosphonic-acid functionalized Ni-cyclam Ni(cycP).^30^ A range of alkanedithiol capping ligands with increasing length (2–8 carbons) separating the two thiol groups (1,2-ethanedithiol (EDT), 1,4-butanedithiol (BuDT), 1,6-hexanedithiol (HexDT) and 1,8-octanedithiol (OctDT)) were employed. The photocatalytic performance was investigated in an aqueous ascorbic acid (AA) solution (3 mL, 0.1 M) under a constant flow of CO_2_ (4 sccm) using automated in-line gas chromatography by irradiating the samples with UV-filtered simulated solar light (*λ* > 400 nm, AM 1.5 G, 100 mW cm^−2^; see ESI† for details). Unexpectedly, we find that the dithiols can activate the bare ZnSe–BF_4_ for enhanced CO_2_RR at conditions similar to the previously optimized system^17^ with a strong dependence on the dithiol length (black trace in Fig. 2 and Table S1†). After 10 h irradiation, a short dithiol (EDT, length (thiol-to-thiol) *ca.* 4.3 Å, molar ratio 100 mol_dithiol_ per mol_QD_) enhances CO_2_RR activity from 0.15 ± 0.03 μmol CO (unfunctionalized) to 0.94 ± 0.19 μmol, whereas longer dithiols (BuDT, HexDT, OctDT, length > 6.8 Å) exhibit a much less distinct effect (CO activity between 0.14 to 0.46 μmol) at a similar loading. In addition, we observe that all the dithiol ligands studied herein inhibit HER significantly without a strong dependence on the dithiol length, leading to enhanced CO-selectivities (Table S1†).

Next, we studied the influence of the same set of dithiols in the presence of the molecular co-catalyst Ni(cycP). While in this case the bare QD surface may still facilitate some CO_2_RR, the 5-fold increase in CO_2_ reduction activity is due to the more efficient molecular catalyst, which acts as the main site for CO_2_RR. In these experiments, the dithiol loading was lowered from 100 to 50 equiv. (mol_dithiol_ per mol_QD_) to allow sufficient space for the molecular catalyst (20 equiv. (mol_Ni(cycP)_ per mol_QD_) and the pH was decreased to 5.5, which was found optimal for ZnSe|Ni(cycP).^7^ Interestingly, the CO_2_RR performance of the hybrid QD-co-catalyst (ZnSe|Ni(cycP)) exhibits a dependence on the employed dithiol. However, unlike the system in the absence of Ni(cycP), the optimum dithiol length for the hybrid QD-co-catalyst is between four and six carbon centers (length from 6.8 Å to 9.3 Å), with a short dithiol such as EDT showing no enhancement in CO production (red trace in Fig. 2). Under optimized conditions, ZnSe|Ni(cycP)|HexDT yields 4.05 ± 0.25 μmol CO, a four-fold enhancement compared to the dithiol-free ZnSe|Ni(cycP), which produces 1.09 ± 0.18 μmol CO. Experiments also reveal that increasing the dithiol length has a nearly linear effect in suppressing HER for ZnSe|Ni(cycP) (Fig. 2A). The reason for the overall higher HER activity in the presence of Ni(cycP) is likely related to (i) the lower dithiol loading (50 equiv. *vs.* 100 equiv. without Ni(cycP)) and (ii) the more acidic pH of 5.5 which renders HER more feasible.

The origin of the evolved CO from CO_2_ was confirmed *via*^13^C-isotopic labelling for the best-performing cases (ZnSe|EDT and ZnSe|Ni(cycP)|HexDT), by running a photocatalytic experiment under an atmosphere of ^13^CO_2_ and analyzing the headspace after reaction *via* FTIR spectroscopy. The change in reduced mass for ^13^C causes the vibration associated with CO to be shifted from 2142 cm^−1^ (^12^CO) to 2095 cm^−1^ (^13^CO; Fig. S4†), which confirms its assignment as ^13^CO.^31^ This observation demonstrates that all the evolved CO originates from CO_2_ and no other carbon sources contribute towards the detected reaction product. Furthermore, no other gaseous or liquid products were found, and no products were evolved in the absence of electron donor, QDs or light, indicating that all components are required for photocatalytic CO_2_RR (Table S2†). The control experiment of ZnSe|dithiol, in the absence of AA, which does not lead to any activity, supports that dithiols do not act as sacrificial electron donors for this particular photocatalyst, despite previous reports that have shown that thiols can act as hole quenchers for other QD-based photocatalysts.^28^

The influence of the marginally different pH and the dithiol loadings employed for both systems (pH 6.5 for ZnSe|dithiol and pH 5.5 for ZnSe|Ni(cycP)|dithiol) was also excluded as the origin of the changes observed in product selectivity. In particular, when the pH is reversed (pH 5.5 for ZnSe|dithiol and pH 6.5 for ZnSe|Ni(cycP)|dithiol), CO production is significantly lower than at the optimal pH conditions, although trends are retained and EDT exhibits the higher activity in the absence of Ni(cycP), while HexDT in the presence of Ni(cycP) (Fig. S5 and Table S3†). When the dithiol loadings are reversed (ZnSe|dithiol at 50 equiv. mol_dithiol_ per mol_QD_) we observe increased HER compared to the optimized conditions and CO formation still peaks with EDT, demonstrating that the changes in product selectivity are not caused by the dithiol loading (Fig. S6†). Increasing the dithiol loading to 100 equiv. in the presence of Ni(cycP) was omitted as the ligand would presumably displace the co-catalyst on the QD surface and lead to lower CO yields, as previously observed with an amine-containing thiol capping ligand.^7^

### Dithiol-QD interactions

**Fig. 3:**
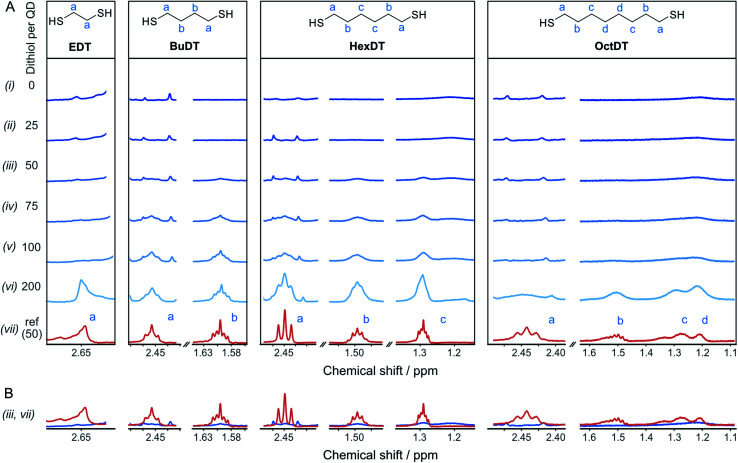
Interactions of dithiols with ZnSe-QDs. (A) ^1^H-NMR spectroscopy titration experiment with aliquots of dithiol (in acetonitrile-d_3_) being added to a suspension of 2 μM ZnSe–BF_4_ QDs in D_2_O. Intensities are not to scale (in-between ligands) and were adjusted for optimal visibility. The spectra were referenced to the residual acetonitrile signal at 1.94 ppm. (B) Overlay of spectra (iii and vii) of the reference ligand spectrum (100 μM) in the absence (orange) and presence of ZnSe (2 μM, blue), respectively, indicating significant suppression and broadening of the ligand signals in the presence of the QD.

To rationalize the influence of the dithiols on the CO_2_RR, the interaction between the different dithiols and the ligand-free ZnSe–BF_4_ QDs were studied in aqueous solution by liquid-phase ^1^H-NMR spectroscopy. The binding of molecules onto the particle surface is reflected in significant broadening of the signals originating from protons near the nanocrystal surface due to their slow and nonuniform tumbling.^17,32–36^ These experiments involved the stepwise addition of defined quantities of dithiol (25, 50, 75, 100, and 200 equiv. (mol_dithiol_ per mol_QD_) per injection, in acetonitrile-d_3_) to a suspension of ZnSe–BF_4_ QDs in D_2_O, leading to the results depicted in Fig. 3. We observe that for quantities of EDT ≤ 100 equiv. per ZnSe–BF_4_ QD, the signals associated to this ligand essentially vanish, which suggests a strong binding affinity of EDT to the QD surface. Only after the addition of 200 equiv. of ligand, a proton signal for bulk EDT appears, which can be assigned to its accumulation in solution. Hence, we conclude that the QD surface can accommodate at least 100 strongly interacting EDT ligands.

On the other hand, signals assigned to BuDT arise in solution from ≥50 equiv. per QD, indicating a lower loading capacity of the QD surface compared to EDT (Fig. 3A). A similar observation is found for HexDT, which may be rationalized by the longer chain length of the two ligands compared to the shorter EDT. It is notable that the ligand signals are significantly broadened in the presence of the QDs compared to a reference spectrum in D_2_O (see below for interpretation). The degree of broadening increases from BuDT to HexDT indicated by the lack of fine structure of the NMR signals, which is most notable for HexDT protons from methylene groups located in the central part of the molecule, denoted as (c) in Fig. 3A.

An overlay of the ligand signal at 100 μM (equivalent to the concentration of 50 ligands per QD) in the absence of QDs allows to assess the reduced signal intensity due to interactions of the ligands with the QDs (Fig. 3B). Proton signals assigned to an OctDT reference spectrum (in D_2_O) appear at ∼100 to 200 equiv. per QD. However, the signals at 1.2–1.3 ppm assigned to the central protons, denoted as (c) and (d) in Fig. 3A, appear at lower molar ratios but overlap with residual solvent signal already present in the QD solution and additionally exhibit strong broadening.

Further in-depth analysis of the ^1^H-NMR spectroscopy titration experiments was performed by integrating the ligand signals (Fig. S7†). BuDT and HexDT follow a near linear increase from 25 to 200 equiv. per QD, whereas OctDT exhibits lower signal intensities overall, which vary depending on the proton signal. For this latter ligand, a very strong increase in intensity is notable from 100 to 200 equiv. per QD for the protons located at the center of the molecule (signal (d), Fig. 3A), which coincides with a substantial broadening of the signals.

The results from the NMR titration experiments suggest the existence of three QD-ligand interaction regimes. In the first regime, the ligands interact very strongly with the QD surface presumably due to covalent binding/H-bonding to the QD surface. Within this regime, the influence of the QD surface on the tumbling of the protons is so strong that the NMR signals essentially vanish.^35,36^ All dithiols tested here show this behavior for dithiol concentrations of ≤25 equiv. per QD, similarly to the previously reported ligand 3-(2-mercaptoethyl)-1-methyl-imidazolium (MEMI).^17^ In the second regime, the signals associated with the ligands are detectable by NMR but are broadened. This broadening indicates that the ligands are in close vicinity of the QD surface which leads to an anisotropic chemical environment for the protons that causes the peaks to broaden – essentially caused by a superposition of many slightly shifted peaks.^35,36^ This broadening increases in the order BuDT < HexDT < OctDT with increasing dithiol hydrophobicity and length. Hence, this regime may be described as a solvation sphere in which weakly interacting ligands accumulate due to hydrophobic interactions with each other and is detectable for BuDT/HexDT/OctDT from >25 equiv. per QD. The strong broadening in the case of OctDT likely causes the overall lower signal intensities because it stretches out over a larger range of chemical shifts, preventing an accurate signal integration. In addition, the lack of well-resolved signals characteristic for ligands in solution at lower loadings (<200 equiv. per QD) may be promoted by the relatively low solubility of the dithiols in an aqueous environment, which leads to their assembly at the QD interfaces through the hydrophobic effect. Furthermore, the intensities of different protons signals of BuDT and HexDT from ≥50 equiv. per QD are nearly identical, indicating that all protons interact within the solvation sphere equally and no orientation is preferred. Finally, in the third regime, ligands accumulate in the bulk solution as evidenced by the sharp signals, which resemble the reference spectrum in the absence of QDs.

### Analogues of alkanedithiols

**Fig. 4:**
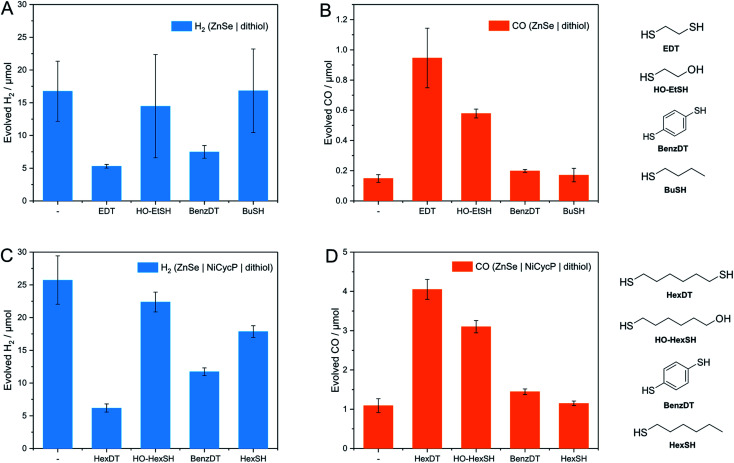
Benchmarking of dithiols with analogous ligands for photocatalytic CO_2_RR. (A and B) Evolved H_2_ and CO in the absence of a co-catalyst: ZnSe|ligand (benchmark EDT) 50 μM ligand, pH 6.5. (C and D) Evolved H_2_ and CO in the presence of a co-catalyst: ZnSe|Ni(cycP)|ligand: (benchmark HexDT) 25 μM dithiol, 10 μM Ni(cycP), pH 5.5. General conditions: AM 1.5G, *λ* > 400 nm, 100 mW cm^−2^, 10 h irradiation, 0.5 μM ZnSe–BF_4_, 0.1 M AA/NaHCO_3_, CO_2_ constant flow (4 sccm), 25 °C.

A series of analogous ligands were studied to explore if (i) the second thiol group is a prerequisite to the enhancement effect and (ii) if this can be explained by the hydrophobic environment introduced through the dithiols. In particular, benzene-1,4-dithiol (BenzDT, length ∼6.4 Å) was chosen as rigid analogue to study if the flexibility of the dithiol ligand is a prerequisite for the enhanced CO activity. While BuDT has a similar length of ∼6.8 Å and exhibits a significant impact on photocatalytic CO_2_RR in the presence and absence of Ni(cycP), BenzDT suppresses HER but only marginally increases CO production, supporting that the flexibility of the dithiol is necessary for the observed enhancement effect in CO_2_RR activity (Fig. 4 and Table S1†). Next, we benchmarked the dithiols against its mercaptoalcohol analogues exhibiting a comparable thiol to hydroxy length to elucidate whether the terminal hydroxy group has any effect. Indeed, ZnSe|1,2-mercaptoethanol (HO-EtSH) enhances CO formation (0.56 ± 0.06 μmol) compared to unfunctionalized ZnSe–BF_4_, and approx. half activity compared to ZnSe|EDT (Fig. 4A and B), but surprisingly, HER is only marginally affected and comparable to unfunctionalized ZnSe–BF_4_. A similar observation was found for ZnSe|Ni(cycP)|1,6-mercaptohexanol (HO-HexSH), which enhances CO evolution notably but does not suppress HER activity (Fig. 4C and D). In the case of monothiols (1-butanethiol (BuSH), 1-hexanethiol (HexSH)), we found that they do not affect the product selectivity compared to unfunctionalized ZnSe–BF_4_ and both HER and CO reduction remain unaffected (Fig. 4).

NMR titration experiments were also extended to the selected analogues of dithiol ligands comprised of only one thiol (monothiol) as well as mercaptoalcohols. HexSH and HO-HexSH were chosen as representatives with six carbon centers and with a comparable length to HexDT. Protons assigned to HO-HexSH are detectable from ≥25 equiv. per QD and increase linearly until 200 equiv. per QD (Fig. S7 and S8†). This finding suggests a weaker affinity for the QD surface compared to HexDT, presumably due to the inability to bind *via* both thiols and the increased hydrophilicity introduced through the hydroxy group. In fact, the NMR peak shape remains well-resolved, suggesting accumulation of ligands in the bulk solution (third regime). The weaker binding of HO-HexSH as compared to dithiols may explain why HER is not as sufficiently suppressed, as reported during photocatalytic CO_2_RR (see above). In contrast, the monothiol equivalent, HexSH, features five distinct signals which appear to various degrees from ≥25 equiv. per QD (Fig. S7 and S8†). Interestingly, terminal protons in closer vicinity to the thiol group (signals denoted as (a), (b), (c) in Fig. S8†) appear later in the ^1^H-NMR spectra (*i.e.*, >100 equiv.) at lower intensities compared to the protons towards the other end of the molecule (signals (d), (e), (f) in Fig. S7 and S8†). This observation confirms that the thiol indeed prefers a conformation with the thiol pointing towards the QD surface. In addition, all signals show distinct broadening similar to that observed for HexDT and OctDT, which increased in accordance with increased hydrophobic character of the dithiol ligands (Fig. 3).

**Fig. 5:**
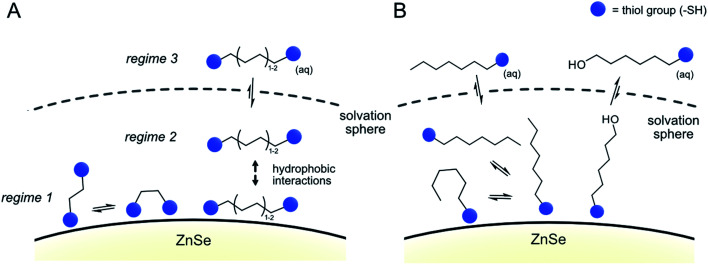
Proposed binding modes of dithiol ligands on the surface of ZnSe QDs. (A) Dithiols and (B) monothiols and mercaptoalcohols.

The data from all described ^1^H-NMR titration experiments support the following types of interactions between the studied ligands and the QD interfaces, clearly showing the differences between mono- and dithiol systems (Fig. 5 and Table 1): the overall lower dynamics of the dithiols in comparison with monothiols suggests that both dithiol –SH groups are involved in the interactions with QDs. This is corroborated by the lower capacity of the QD surface for the longer dithiols (BuDT, HexDT) than for the shorter EDT, suggesting that both –SH groups of the longer dithiols interact with QD surfaces and thereby occupy more space in a bidentate configuration rather than in a dangling/monodentate configuration. The bidentate binding mode of BuDT/HexDT is further supported by the observation that in the NMR titration experiments, the signals for all protons in the alkane backbone are increasing with equal intensities (Fig. S7A†). In contrast, for the monothiol (HexSH) the intensities of proton signals from terminal methylene and methyl groups increase faster in comparison with the signals from methylene groups located in the close proximity to the anchoring thiol, indicating that the monothiol binds in a preferred orientation with the terminal proton facing into the solution (Fig. S7B†). Note, these observations are indirect indications of a bidentate binding mode of dithiols but do not preclude the existence of a monodentate binding mode because NMR-spectroscopy is essentially *blind* towards the strongly interacting ligands in regime 1. Dithiols (C_4+_) and monothiol HexSH bind to the QD surface strongly and after a saturation point accumulate within the solvation sphere and thereby introduce a significant degree of hydrophobicity on the QD interfaces. In addition, HO-HexSH interacts with the QD surface in a weaker manner when compared to dithiols and the terminal hydroxy group is likely to stretch into solution interacting with surrounding water molecules. This is turn limits the hydrophobic character of the solvation sphere of the QD/HO-HexSH hybrid, lowering the number of interacting ligands, affecting both HER suppression and CO_2_RR.

**Table 1:** Summary of interactions of dithiols (and analogues) with ZnSe QDs

Ligand	Class	Length^a^/Å	No. of strongly interacting ligands
EDT	Dithiol	4.3	100
BuDT	Dithiol	6.8	25–50
HexDT	Dithiol	9.3	25–50
OctDT	Dithiol	11.7	50–100
HexSH	Monothiol	∼9	25–50
HO-HexSH	Mercaptoalcohol	9.3	<25

a Estimated by measuring the distance from the terminal thiol to the other end of the molecule in its stretched conformation.

We next considered the possibility of dithiols to cross-link individual particles leading to larger aggregates, as reported previously.^37^ Dynamic light scattering experiments suggest that all ligands employed here (dithiols, HexSH, HO-HexSH) facilitate some degree of agglomeration after saturation of the surface with strongly interacting ligands (>25 equiv. per QD), which is most significant for EDT (particles size ∼250 nm) compared to unfunctionalized ZnSe–BF_4_ (∼10 nm) (Fig. S9A and B†). Nevertheless, we find that the presence of a large excess of AA already leads to much larger agglomerates of ∼1600 nm regardless of any capping ligands (Fig. S9C†). Thus, the influence of the dithiol ligands is negligible compared to that of AA and we therefore believe that the different ligands do not result in performance differences based on aggregation during photocatalysis. In addition, dithiols were found to not affect the photophysics of the QDs, as confirmed by the recorded steady-state UV-vis absorption and photoluminescence (PL) spectra, which remain unchanged in the absence and presence of dithiols (Fig. S10†).

## Discussion

The results from NMR titration experiments and DLS support that the effects observed during photocatalysis could stem from multiple physicochemical effects. Because the UV-vis/PL profiles remain unchanged in the presence of dithiols, effects on the QD steady-state photophysics are discarded. NMR experiments demonstrate that dithiols can introduce a hydrophobic environment (increasing with the dithiol length) which could regulate substrate access and may provide a favorable microenvironment for CO_2_RR. Implications on the charge transfer dynamics cannot be excluded at this point and were observed for similar particle-ligand systems,^38^ but should generally lead to lower electron transfer rates to acceptor molecules with longer ligands, which contrasts the photocatalytic results obtained in this work. The hydrophobic environment may explain a suppression of HER and enhanced CO_2_RR due to a lower local water concentration and increased CO_2_ concentration. However, this hypothesis fails to explain why no enhancement effect is observed for BuDT/HexDT on the bare ZnSe surface, unless the effect is related to the lower capacity of the QDs for BuDT/HexDT. The local hydrophobic environment also falls short of rationalizing the lack of influence of monothiols (BuSH/HexSH), which do not enhance CO_2_RR, even though they provide a hydrophobic environment. The fact that they also do not suppress HER is surprising. On the other hand, mercaptoalcohols enhance CO_2_RR to a lesser extent than dithiols, but still significant compared to non-functionalized QDs. Their reduced capacity to suppress HER, however, is a further argument against hydrophobic effects as the sole explanation because the hydroxy group essentially removes hydrophobic interactions. Finally, particle agglomeration is considered an unlikely contribution to the observed trends, because even though all ligands tested form some aggregates (<250 nm) (in particular EDT), this is small in comparison to the effect of the electron donor AA, which leads to much larger aggregates of ∼1600 nm.

Overall, the results point towards a more unique effect of the second thiol group in the local chemical environment of the CO_2_RR site on the QD surface which influences both HER and CO_2_RR. Hence, we turned to DFT simulations to explore if the dithiols may affect CO_2_RR in the secondary coordination sphere of the catalytic site through non-covalent interactions (NCIs) with the reaction intermediates.

## DFT calculations

**Fig. 6:**
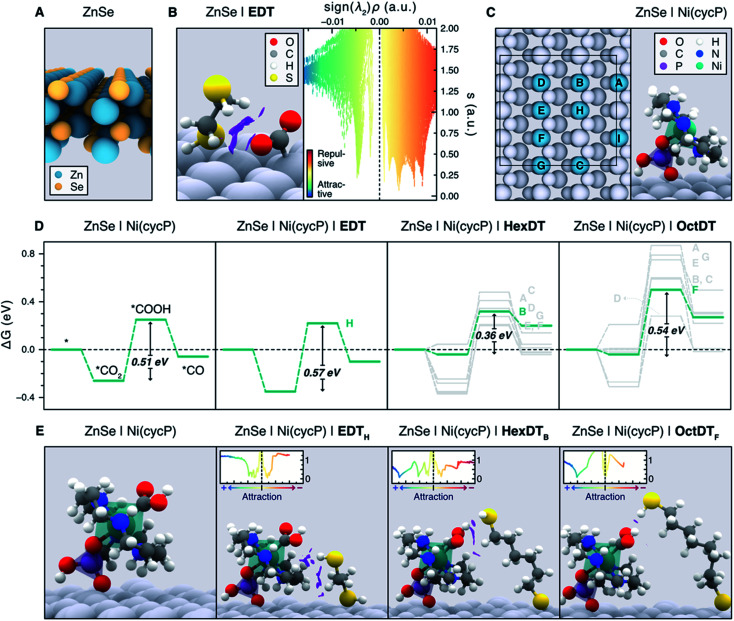
(A) Side view representation of the modelled ZnSe(220) surface slab. (B) Activated *CO_2_ intermediate atop a surface Zn site on ZnSe|EDT (left), displaying the non-covalent interactions (NCIs) as purple isosurfaces (isovalue = 0.4 a.u.). NCIs are represented pseudo-quantitatively on the right panel with a plot of the reduced density gradient (*s*) as a function of the electron density multiplied by the sign of the second eigenvalue of the Hessian matrix (sign (*λ*_2_)*ρ*), which effectively displays the NCIs as distinct peaks. Colder and warmer colors depict attractive and repulsive interactions, respectively. (C) Representation of all the surface Zn sites (*A*–*I*) considered for the adsorption of Ni(cycP) and the investigated dithiols (left). Note the Ni(cycP) cocatalyst was adsorbed on the site *I* and that only the sterically accessible sites *A*–*H* were considered for ligand adsorption. The right panel depicts the side view of the ZnSe|Ni(cycP) resting state used in the mechanistic studies. (D) Gibbs energy diagrams for the CO_2_RR on the bare ZnSe|Ni(cycP) and in the presence of a monodentate EDT, HexDT and OctDT ligand adsorbed on all the sterically accessible sites *A–H* (see labels beside the highest energy point). Gibbs energies are also provided in Table S7.† The most energetically favorable pathway for each functionalized system is highlighted in green. (E) Side view representation of the *COOH intermediate on the bare ZnSe|Ni(cycP) and the lowest-energy functionalized systems (green lines in D). NCI isosurfaces (isovalue = 0.4 a.u.) and plots (insets) are also shown. In B, C and E, surface atoms are greyed out for clarity.

Unveiling the nature of the interactions between the dithiol capping ligands and ZnSe QDs, as well as their influence on CO_2_RR, is critical to drive the discovery of more efficient photocatalysts for this process. Experimental observations point towards the length and flexibility of dithiols as the main factors facilitating CO_2_ activation and its subsequent reduction, either *via* a surface promoted mechanism, or a pathway enabled by the immobilized Ni(cycP) co-catalyst (see Fig. 1). Based on our recent findings on MEMI-functionalized ZnSe-QDs for CO_2_RR,^17^ we posited that NCIs between the capping dithiol ligands and CO_2_ might play a key role when non-binding thiol moieties are neighboring the second coordination sphere of the catalytic active site. In particular, we envisioned that the positive influence of the dithiols is maximized in the surface promoted mechanism when shorter ligands are used, as they can interact more strongly with the CO_2_RR intermediates adsorbed on the QD surface. In contrast, longer-reaching ligands are deemed to be better suited to stabilize the CO_2_RR intermediates in the co-catalyst promoted pathway, further away from the QD surface. To confirm these hypotheses, and assess the ability of dithiol ligands to suppress the competing HER, we carried out an exhaustive computational investigation by means of periodic DFT calculations using the Perdew–Burke–Ernzerhof (PBE) functional with Grimme's D3 dispersion corrections (see ESI† for details). To describe the bare ZnSe QD, we used the cubic ZnSe bulk structure shown in Fig. S11† to construct the 4-layer ZnSe(220) surface slab depicted in Fig. 6A (see Computational Methods in the ESI† for details), as this structure has been previously shown to accurately represent the morphology of the system.^17^

To shed light on the influence of the dithiols length on the surface promoted pathway, we performed DFT calculations on functionalized ZnSe(220) slab models using the surface concentrations observed in the NMR titration experiments for shorter (EDT) and longer (HexDT and OctDT) ligands (Table S4†). The lowest energy configuration for each of these systems corresponds to one EDT, HexDT and OctDT ligand adsorbed in a bidentate mode on the Zn sites of a ZnSe(220) surface with periodicities *p* (1 × 1), *p* (3 × 1) and *p* (1 × 3), respectively. Using the structures for the bare ZnSe and ZnSe|nDT (nDT = EDT, HexDT, OctDT) systems, we subsequently investigated their ability to activate CO_2_ with and without a photogenerated electron, which is believed to be the first step in photocatalytic CO_2_RR.^39^ Interestingly, of all the studied systems, only ZnSe|EDT in the presence of a photogenerated electron was able to activate *CO_2_ atop a surface Zn site (Fig. 6B, left), which was found to be the main active site for both CO_2_RR and HER (see below). Note *CO_2_ activation on the bare ZnSe-QDs surface, ZnSe|HexDT, and ZnSe|OctDT was not achieved, and resulted in CO_2_ being released back into the gas-phase.

The switch from the bidentate to the monodentate binding mode is a prerequisite for CO_2_ activation. This is in line with our calculations (Table S4†), which show that EDT requires the least energy for switching from a bidentate to a monodentate configuration (*i.e.* 0.15 eV compared to 0.42 and 0.17 eV for HexDT and OctDT, respectively). On the contrary, longer dithiols are, in general, more flexible and present less repulsion with the surface in a bidentate configuration, making the change to the monodentate mode more energetically demanding. We also note that all attempts to activate *CO_2_ without the photogenerated electron resulted in CO_2_ desorbing away from the surface. The NCI analysis between EDT and the activated *CO_2_ (Fig. 6B, right) reveals that the stabilization of this intermediate is mainly governed by H-bonding between the thiol group and *CO_2_ and a set of repulsive steric interactions that tie the CO_2_ molecule to the surface. The exceptional ability of EDT to stabilize this first CO_2_RR intermediate is in good agreement with experimental observations, which show a 6-fold (3.5-fold) increase in CO production with ZnSe|EDT compared to the bare surface and ZnSe|HexDT (ZnSe|OctDT).

To assess the influence of the dithiols length on the HER activity, we next modelled the Gibbs adsorption energy of a H atom on the surface of the functionalized QD systems as a descriptor for this process.^40,41^ Our calculations indicate that the remaining surface Zn atoms in the presence of a photogenerated electron are the main HER active sites, exhibiting nearly thermoneutral Δ*G*_H_ values of −0.28 eV, 0.26 eV and 0.06 eV on ZnSe|EDT, ZnSe|HexDT and ZnSe|OctDT, respectively. Hence, HER is predicted to occur on all the functionalized systems to a similar extent, in agreement with experiments (Fig. 2A). Note that the subtle differences observed in H_2_ production with the various dithiols may be attributed to variabilities in their surface coverages and lengths. More specifically, small dithiols can cover the QD surface more efficiently – particularly EDT, whose length almost matches the interspacing between Zn atoms. On the other hand, longer dithiols such as HexDT and OctDT do not cover all the HER sites, although their accessibility is reduced with ligand length. Taken altogether, and the stronger binding of dithiols compared to H, we can rationalize the trends in HER activity observed experimentally (Fig. 2A, black trace), *i.e.* ZnSe ≫ ZnSe|HexDT > ZnSe|EDT ≈ ZnSe|OctDT.

Having elucidated the role of the dithiol ligands in the surface promoted pathway, we set out to explore their influence on the co-catalyst promoted mechanism on ZnSe|Ni(cycP) (see Fig. 1). In this case, the experimental coverage of Ni(cycP) was reproduced by modelling a *p* (3 × 2)-ZnSe(220) surface containing one co-catalyst molecule adsorbed on the surface Zn site *I* (Fig. 6C) *via* a singly deprotonated phosphonate group and two dithiol ligands, according to the experimental pH of 5.5. From this structure, all possible different configurations arising from the adsorption of two EDT, HexDT and OctDT ligands atop the remaining 8 Zn sites (sites *A* to *H* in the left panel of Fig. 6C) were considered in the presence of a photogenerated electron, leading to a total of 1 (1), 7 (11) and 7 (6) monodentate (bidentate) configurations, respectively (see Table S5†).

The CO_2_RR mechanism with a photogenerated electron was then investigated with and without the presence of capping ligands, resulting in the Gibbs energy diagrams presented in Fig. 6D. As in the surface promoted pathway, the reaction begins with the activation of CO_2_, this time on the Ni center of the co-catalyst, followed by two consecutive proton-coupled electron transfer steps that yield *COOH and eventually H_2_O and *CO. In the absence of dithiol ligands (ZnSe|Ni(cycP)), calculations indicate that the formation of *COOH is the only endergonic step, rendering this process as the most likely rate determining step with a Gibbs energy change of +0.51 eV. Based on this result, and the fact that CO_2_ cannot be stabilized without the presence of a dithiol ligand, we conclude that ZnSe|Ni(cycP) can promote CO_2_RR more efficiently, which is supported by the 7-fold increase in CO production obtained experimentally with ZnSe|Ni(cycP) compared to the bare ZnSe-QD.

When assessing the same mechanism for the different configurations with the functionalized systems, ZnSe|Ni(cycP)|nDT, we observed that the binding energies of the CO_2_RR intermediates are only influenced by the presence of the mono-dentate dithiol adsorbed in the vicinity of the co-catalyst. This is because the distance between the adsorbed mono- and bidentate dithiols is *ca.* ≥ 4.0 Å (see Table S6†), and therefore, the presence of bidentate ligands does not affect the energetics of the reaction intermediates. Hence, we investigated the CO_2_RR mechanism with only the monodentate dithiol and for those configurations wherein the non-coordinated thiol group could interact with the CO_2_RR intermediates adsorbed on Ni(cycP). We note that, even though the bidentate mode is the most stable configuration for all the considered dithiols, the energy difference between this mode and the monodentate one is sufficiently small (*ca.* 0.15–0.40 eV, Table S4†) to consider the likely existence of a subset of monodentate ligands at room temperature. Such a subset would be difficult to detect through the NMR titration experiments conducted above. Furthermore, this monodentate configuration explains the ligand length dependence on the CO_2_RR activity observed in experiments, as we describe in the following. Due to the short nature of EDT, the interaction with the coordination sphere of Ni(cycP) was only possible for the monodentate ligand on the Zn site *H* (ZnSe|Ni(cycP)|EDT_H_), leading to binding energies of −0.35 eV, 0.22 eV and −0.10 eV for *CO_2_, *COOH and *CO, respectively (Fig. 6D, second leftmost panel). Again, the most endergonic step was found to be the formation of *COOH with a very similar energy than that of the unfunctionalized system (0.57 *vs.* 0.51 eV), indicating that EDT does not influence the baseline activity of ZnSe|Ni(cycP) due to its incapacity to effectively interact with the CO_2_RR intermediates in the co-catalyst promoted pathway. This was confirmed by NCI analysis, which reveals that the bulk of the interactions between EDT_H_ and Ni(cycP) is mainly constituted by steric effects between the hydrocarbon chain of the ligand and the base of the co-catalyst (Fig. 6E, second left panel). Therefore, the enhanced CO production observed experimentally with ZnSe|Ni(cycP)|EDT compared to ZnSe|Ni(cycP) can be attributed to the ability of EDT to favor the surface-promoted pathway, as observed in our calculations, while the decrease in HER is due to the reduced number of HER-active Zn sites available on the surface, which are covered by EDT.

For HexDT and OctDT we found a total of 7 distinct monodentate configurations that can interact with the CO_2_RR intermediates on ZnSe|Ni(cycP), leading to the reaction profiles shown in the two right panels of Fig. 6D. Among these configurations, the ones exhibiting the least endergonic formation of *COOH from *CO_2_ (highlighted in green) correspond to ZnSe|Ni(cycP)|HexDT_B_ and ZnSe|Ni(cycP)|OctDT_F_, with energy changes of +0.36 and +0.54 eV, respectively. Importantly, these results indicate that HexDT has the optimal length to interact more effectively through the dangling thiol *via* H-bonding with the CO_2_RR intermediates adsorbed on the co-catalyst, which reduces the energy of the most endergonic step by 0.15 eV compared to ZnSe|Ni(cycP). Indeed, NCI analysis shows that HexDT exhibits overall a set of more favorable interactions with the *COOH intermediate compared to EDT and OctDT (Fig. 6E). We note that the latter two ligands display similar energetics than ZnSe|Ni(cycP), suggesting that EDT (OctDT) is too short (long) to efficiently interact with the CO_2_RR intermediates in the co-catalyst promoted pathway.

## Conclusions

In summary, we report a surface modification strategy for ZnSe QDs based on dithiols that promotes photocatalytic CO_2_RR in the absence and presence of an additional molecular co-catalyst, depending on the dithiol length. The dithiol–QD interactions have been studied quantitatively using ^1^H-NMR spectroscopy, allowing the determination of the number of strongly interacting ligands and revealing a solvation sphere dominated by hydrophobic interactions for the longer dithiols (C_4+_). Photocatalytic studies using ZnSe–BF_4_ QDs in aqueous ascorbate solution show that EDT activates the QDs for CO_2_RR accompanied by a reduction of the HER activity compared to the bare QD surface. In the presence of the molecular co-catalyst, we show that a longer dithiol such as HexDT further accelerates CO_2_RR while suppressing HER. A series of control experiments employing monothiols and mercaptoalcohols render the hydrophobic effects unlikely as sole explanation of the observed changes during photocatalysis. DFT calculations provide a framework to rationalize the length dependent influence during photocatalysis, showing that EDT has the suitable length to stabilize the key *CO_2_^*δ*−^ intermediate on the QD surface through H-bonding, promoting a surface-mediated mechanism. In contrast, the length and flexibility of HexDT allows to stabilize more efficiently the endergonic formation of *COOH on the Ni(cycP) co-catalyst. DFT calculations demonstrate that CO_2_RR *via* a surface or co-catalyst-promoted pathway can be ‘switched’ on and off depending on the length of the dithiol. In addition, calculations illustrate that both CO_2_RR mechanisms require the binding of the dithiol ligand in a monodentate configuration in order to stabilize the key reaction intermediates through the dangling thiol group. This explains why experiments with HO-EtSH and HO-HexSH lead to an enhanced CO_2_RR activity while monothiols do not influence product selectivity as these ligands cannot stabilize the CO_2_RR intermediates and do not cover efficiently the HER active sites due to their monodentate configuration. Hence, we conclude that ideal capping ligands for CO_2_RR can have the ability to coordinate bidentately to the surface to block the HER active sites but should have the ability to turn monodentate to stabilize the relevant CO_2_RR intermediates. In future work, we envision to expand the insights gained herein to other semiconductor systems as well as investigating polydentate ligands. Overall, this work presents dithiol capping ligands as a novel tool to manipulate photocatalytic CO_2_RR and steer the product selectivity between HER and CO_2_RR on colloidal nanoparticles.

## Data availability

Raw data related to this article are available at https://doi.org/10.17863/CAM.83674 (experimental data) and https://doi.org/10.19061/iochem-bd-6-131 (computational data).

## Author contributions

C. D. S., M. G.-M. and E. R. designed the project. C. D. S. prepared and characterized the ZnSe particles and conducted photocatalytic experiments. A. C. and E. M.-T. performed the DFT studies. K. S., V. B. and C. D. S carried out NMR titration experiments. C. D. S. collected UV-vis and photoluminescene spectra. C. D. S. and K. S performed DLS titrations. G. N. and A. J. C. prepared Ni(cyclamP). All authors analyzed the data, discussed the results and assisted with the manuscript preparation.

## Conflicts of interest

The authors declare no conflict of interests.

## Supplementary Material

SC-013-D2SC00890D-s001
