# Supplementary material for: Tuning the local chemical environment of ZnSe quantum dots with dithiols towards photocatalytic CO2 reduction
Source: Chem Sci. 2022 Apr 11;13(20):5988–98. doi: 10.1039/d2sc00890d (PMC9132019; doi:10.1039/d2sc00890d)
Supplement: SC-013-D2SC00890D-s001 [file SC-013-D2SC00890D-s001.pdf]

Electronic Supplementary Material (ESI) for Chemical Science.
This journal is © The Royal Society of Chemistry 2022

Electronic Supplementary Information

Tuning the local chemical environment of ZnSe QDs with dithiols towards photocatalytic CO_2 reduction

Constantin D. Sahm[1], Anna Ciotti[2†], Eric Mates-Torres[2†], Vivek Badiani[1], Kamil Sokołowski[1], Gaia Neri[3], Alexander J. Cowan[3], Max García-Melchor[2*] and Erwin Reisner[1*]

[1] *Yusuf Hamied Department of Chemistry, University of Cambridge, Lensfield Rd, Cambridge, CB2 1EW, United Kingdom*

[2] *School of Chemistry, CRANN and AMBER Research Centres, Trinity College Dublin, College Green, Dublin, 2 Ireland*

[3] *Stephenson Institute for Renewable Energy, Department of Chemistry, The University of Liverpool, Crown Street, Liverpool L69 7ZD, UK*

Corresponding authors: garciamm@tcd.ie, reisner@ch.cam.ac.uk

[†] *These authors contributed equally to this work.*

Materials. Zinc stearate (purum, Sigma-Aldrich), octadecene (90% techn., Sigma-Aldrich), selenium powder (99%, Sigma-Aldrich), *n*-heptane (99%, Aldrich), methanol (99.8%, Fisher-scientific), acetone (laboratory reagent grade, Fisher-scientific), 1-butanol (99%, Alfa Aesar), trimethyloxonium tetrafluoroborate (96%, Sigma-Aldrich), L-ascorbic acid (99%, Sigma-Aldrich), 1,2-ethanedithiol (98%, Sigma-Aldrich), 1,4-butanedithiol (97% Sigma-Aldirch), 1,6-Hexanedithiol (97%, Acros), 1,8-octanedithiol (99%, Acros), 1-Butanethiol (99%, Sigma-Aldrich), 1-Hexanethiol (96%, Acros), 2-Mercaptoethanol (99%, Sigma-Aldrich), 6-Mercapto-1-hexanol (97%, Sigma-Aldrich) were used as received. Anhydrous solvents were purchased from Acros Organics with the following purities: $CHCl_3$ (99.9%), *N*,*N*-dimethylformamide (DMF, 99.8%). All aqueous experimental solutions were prepared with ultrapure water (DI water; Milli-Q®, 18.2 MΩ cm). $^{13}CO_2$ (>99 atom% ^{13}C) was purchased from Sigma Aldrich. Ni(cycP) was prepared as previously reported.[1]

Powder X-ray diffraction (XRD). XRD was conducted using an X'Pert PRO by PANalytical BV instrument using CuK_α irradiation.

Infrared spectroscopy. Gas-phase transmission IR spectra of the photoreactor headspace were recorded on a Thermo Scientific Nicolet iS50 FT-IR spectrometer in transmission mode.

Transmission electron microscopy (TEM). TEM images were collected using a Thermo Scientific (FEI) Talos F200X G2 TEM, operating at an accelerating voltage of 200 kV. Samples were prepared by drop-casting a dilute QD-solution on holey-carbon coated Cu grids followed by evaporation of the solvent.

UV−Vis spectroscopy. UV−Vis spectra were recorded on an Agilent Cary 60 UV−Vis spectrophotometer using quartz glass cuvettes (1 cm path length).

Photoluminescence (PL) spectroscopy. PL spectra were recorded on an Edinburgh Instruments FS5 Spectrofluorometer using a Suprasil Quartz (QS) cuvette with 1 cm path length at room temperature.

Dynamic light scattering (DLS). DLS measurements of ZnSe-BF_4 (0.5 µM in water (neutral pH) or AA, pH adjusted to 5.5) were conducted using a Malvern Zetasizer Nano ZS90 instrument at 25 °C.

^{1}H Nuclear magnetic resonance (NMR) titration experiments. ^{1}H-NMR spectra were recorded on a Bruker AVANCE 500 with a TCI Cryoprobe system (500 MHz). Chemical shifts are recorded in D_2O in ppm with the internal reference set to the residual acetonitrile-d3 peak at δ = 1.94 ppm. In a typical NMR titration experiment, 0.5 mL of a 2 µM ZnSe-QD solution in D_2O is mixed with specific amounts of capping ligand (dithiol or dithiol analogue) stock solution (typically 10 µL of a 2.5 mM in acetonitrile-d) inside an NMR tube under an atmosphere of N_2. All NMR titration spectra were measured with 256 scans.

Synthesis and characterization of ZnSe QDs. Ligand-free ZnSe-BF_4 QDs were prepared as reported previously.[2] The mean particle size was determined from transmission electron microscopy images (d = 4.5±0.7 nm) and the particles feature a good visible light absorption onset (λ_{max} = 416 nm). To calculate the QD concentration in the stock solution, the Zn^{2+} and Se^{2-} concentration determined by ion-coupled plasma optical emission spectroscopy, was divided by the number of Zn atoms per QD based on the mean particle diameter and the bulk density of ZnSe (5.262 g cm^{-3}). The full characterization of the QDs can be found in Figure S1.

Photocatalytic CO_2 reduction

Sample preparation. A ZnSe-BF_4 stock solution (64.1 μM in DMF, 23.40 μL), a capping ligand solution (5.0 mM in DMF, typically 30 μL) followed by co-catalyst solution (Ni(cycP, 5 mM in H_2O) were added stepwise to a Pyrex glass photoreactor (Chromacol 10-SV, Fisher Scientific) containing a magnetic stirrer bar. The mixture was diluted with ascorbic acid (AA, 0.1 M in water, pH adjusted to 7 with NaOH) to a total solution volume of 3 mL. In the absence of Ni(cycP), $NaHCO_3$ powder (25 mg) was further added to increase the pH to 8.3. The photoreactor was then sealed with a rubber septum and pierced with two needles (inlet and outlet).

Constant flow-setup with automated product quantification. The inlet of the photoreactor was connected to a Mass Flow Controller (Brooks GF040) supplying a stream of CO_2 (CP Grade, BOC, humidified with a water bubbler) with a flow rate of 4.0 sccm. The flow rate at the GC outlet was verified prior to the experiment with an Alicat gas flow meter to avoid gas leakage. The outlet of the photoreactor was connected to a flow selection valve controlled by a Shimadzu Tracera GC-2010 Plus gas chromatograph for product quantification of the gaseous reaction products (see below). Six samples (two triplicates of identical conditions) were typically analyzed in parallel. Upon purging with a constant stream of CO_2, the solution pH decreased to 6.5 (absence of Ni(cycP)) or 5.5 (presence of Ni(cycP) due to saturation with CO_2. The photoreactor was purged for a further 45 min in the dark and sampled via online GC quantification. The first two injections of each sample were used to determine a "background" peak which was subtracted from further injections. The photoreactor was then placed in a water bath maintained at 25 °C, stirred and irradiated by a solar light simulator (Newport Oriel, AM 1.5G, 100 mW cm^{-2}). The six samples were evenly distributed within the light simulator to account for possible variations of the light intensity depending on the position in the simulator. UV irradiation was filtered with a 400 nm cut-off filter (UQG).

For ^{13}C isotopic labelling, photocatalysis experiments were performed as described above, but with accumulating products in the headspace under steady-state conditions and using $^{13}CO_2$ as the headspace gas. After 1000 min (16.7 h), the photoreactor headspace was transferred to an evacuated gas IR cell (SpecAc, 10 cm path length, equipped with KBr windows) and a high-resolution gas-phase transmission spectrum was collected.

The Shimadzu Tracera GC-2010 Plus gas chromatograph (GC) used a barrier discharge ionization detector, kept at 300 °C, and was equipped with a Hayesep D (2 m * 1/8'' OD * 2 mm ID, 80/100 mesh, Analytical Columns) pre-column and a RT-Molsieve 5A (30 m * 0.53 mm ID, Restek) main column in order to separate H_2, O_2, N_2, CH_4 and CO while hindering CO_2 and H_2O to reach the Molsieve column. He carrier gas (Grade 5.0, BOC) was purified (HP2-220, VICI) prior to entering the GC. The column temperature was kept at 85°C. The gaseous flow from the flow selection valve was passed through a loop (volume 1.0 mL) and injected approximately every 4.25 min into the GC. Effectively, each individual sample was injected every 25.5 min. The GC calibration was performed with a known standard for H_2, CO and CH_4 (2040 ppm H_2/2050 ppm CO/2050 ppm CH_4 in balance gas CO_2, BOC) by diluting the mixture with pure CO_2.

Data analytics. The data was processed and visualized using the statistical programming language *R* with the *tidyverse* library.[3,4] First, the flow rates were corrected by subtracting a "background" peak obtained in the dark prior to irradiation (we noticed a marginal CO background peak depending on the residual amount of oxygen present in the sample stream – a feature of the gas chromatograph and not the sample). Second, the momentary product evolution rate corresponding to each injection was calculated using the following formula. ˙

$$product\ evolution\ rate = \frac{p * \dot{V} * \frac{Area\ GC}{f_i}}{R\,T}$$

where p is the pressure in the photoreactor (ambient pressure, 101325 Pa), is the flow rate (4.0 sccm), R is the universal gas constant, T is the temperature (298 K) and f_i is the response factor for each gas determined by the calibration procedure. Third, the total amount of evolved product was calculated using trapezoidal integration of the product evolution rates. The three independent replicates of identical conditions were averaged by calculating the mean and standard deviation over irradiation time and sample. For visual display, the actual values for each sample are plotted as transparent scatter, whereas the mean is represented as a smoothened continuous line. In addition, the standard deviation is visualized by the shaded area surrounding the mean where the transparency is proportional to the standard deviation. Specifically, the calculated standard deviation is used to compute a Gaussian density for that standard deviation, plotting a cloud with the opacity proportional to the density. This appears as a vertical "cloud" of uncertainty[5]. The maximum of the uncertainty cloud is set to 1 standard deviations.

Computational methods

Periodic density functional theory (DFT) calculations reported in this work were carried out using the Perdew-Burke-Ernzerhof (PBE) functional[6] with dispersion corrections through the zero-damping DFT-D3 method by Grimme,[7] as implemented in the Vienna *Ab Initio* Simulation Package (VASP) software, version 5.4.4.[8] The core electrons of the Zn, Ni, Se, S, N, O, C, P and H atoms were described by projector-augmented wave (PAW) pseudopotentials,[9] while their valence electrons were expanded in plane waves with a kinetic energy cut-off of 500 eV. Geometry optimizations were performed with an energy convergence criterion for the electronic steps of 10^{-6} eV and a force criterion of 0.015 eV/$Å^2$ for the ionic steps, using a Gaussian smearing with a width of 0.05 eV.

The ZnSe cubic bulk structure (space group $F\bar{4}3m$), displayed in Figure S11A, was retrieved from the Materials Project database (mp-1190).[10] The equilibrium lattice constant was determined by optimizing the structure with lattice parameters ranging ±5% of the initial value and fitting the energies to the Birch-Murnaghan equation of state, while sampling the reciprocal space with Γ-centered k-point grids of 3×3×3, 5×5×5, 7×7×7, 9×9×9 and 11×11×11. Following a convergence criterion of 1 meV $atom^{-1}$, the 5×5×5 k-point grid corresponding to a density of 28.7 points $Å^{-1}$ was selected for surface calculations; this bulk displayed a band gap of 1.17 eV (Figure S11B), which is in good agreement with the values predicted theoretically at similar levels of theory.[11,12] To describe the QD surface, we employed the 4-layered (200) slab model reported in our previous work[1] with a vacuum space of 15 Å, wherein the two bottom layers were fixed to their bulk positions and the two topmost layers were allowed to relax. Gas molecules were optimized at the Γ-point and with 15 Å of vacuum along the three axes.

Gibbs energy corrections to the electronic energy were computed with the ASE thermochemistry module[12] at the temperature of 298 K and pressure of 1 atm. For gas-phase molecules, these corrections were calculated via the ideal gas limit method, which include translational, rotational, and vibrational contributions. For adsorbates, only vibrational contributions treated harmonically were considered. For the adsorbed Ni(cycP) cocatalyst and dithiol ligands, Gibbs corrections were determined in the systems ZnSe | Ni(cycP) and ZnSe | DT, respectively, and then applied to the combined system ZnSe | Ni(cycP) | DT. This approach was validated by computing the Gibbs corrections for EDT in ZnSe | Ni(cycP) | EDT, where EDT was monodentately adsorbed on site **H**, or bidentately on sites **H**-**B** (see Figure 6C), finding energy differences of only 0.01 eV compared to the energies obtained with the system ZnSe | EDT. Similarly, the Gibbs corrections for the CO_2RR intermediates on the Ni(cycP) cocatalyst in ZnSe | Ni(cycP) | DT were adopted from the system without the adsorbed dithiol. The validity of this approach was confirmed by computing the corrections for the $*CO_2$ intermediate in the system Ni(cycP) | ZnSe and comparing them with those calculated in ZnSe | Ni(cycP) | EDT, obtaining energy differences which are deemed negligible (< 1 meV).

Non-covalent interactions (NCIs) were assessed by taking the geometry of the adsorbed species optimized in VASP and computing their electron density via single-point calculations with the dispersion-corrected hybrid exchange-correlation functional ωb97xd[13] and the Gaussian09 software, Revision E.01.[14] In these calculations, Ni atoms were described with the effective core potential Lanl2dz and an additional *f*-polarization function (exponent = 3.130),[15] while the 6-31g(d,p) basis set was used to describe the C and H atoms. The latter basis set with an additional *p*-diffuse function was employed to represent the electrons of the more electronegative S, N, P and O atoms. Based on the electron density ρ, the reduced density gradient $s(\rho)$ was calculated according to Eq. 1 using the software Critic2.[16]

$$s(\rho) = \frac{1}{2(3\pi^2)^{1/3}} \frac{|\nabla\rho|}{\rho^{4/3}} \qquad (1)$$

We note that at regions far from the molecule, both ρ and $s(\rho)$ approach zero exponentially. Similarly, $s(\rho)$ approaches zero at regions where NCIs are present, and becomes identically zero at the covalent bonds, *i.e.* critical points of ρ. To distinguish between attractive and repulsive interactions, the Laplacian of the density, $\nabla^2\rho$, is computed. For repulsive interactions, ρ is expected to be a minimum, and therefore, all components of $\nabla^2\rho$ along the 3 dimensions are positive. On the other hand, for attractive interactions, ρ is characterized by a maximum along one direction whose eigenvalue in the Hessian matrix of ρ is called λ_2. NCI-plots are constructed by plotting $s(\rho)$ against ρ multiplied by the sign of λ_2, sign(λ_2)ρ. These plots allow to distinguish between bonded ($\lambda_2 < 0$) and non-bonded ($\lambda_2 > 0$) NCIs, while the value of ρ indicates the strength of these interactions.[17]

Assessment of the ZnSe-QD surface coverage

To model the adsorption of the dithiol ligands and the Ni(cycP) cocatalyst, the ZnSe-QDs coverage was calculated, based on the experimental concentration of species on the QD surface according to the equation:

$$\frac{n°_{ligands}(QD)}{4\pi r_{QD}^2} = \frac{n°_{ligands}(unit\ cell)}{a \times b} \qquad (2)$$

where r_{QD} (2.27 nm) is the experimental radius of the QDs, and a (5.75 Å) and b (4.06 Å) are the lattice constants of the modelled surface unit cell.

For EDT, the experimental coverage for the system ZnSe | EDT corresponded to a single molecule adsorbed in a $p(1\times1)$ unit cell, while for HexDT and OctDT this corresponded to a single molecule in a $p(1\times3)$ and $p(3\times1)$ unit cell, respectively. In the systems ZnSe | Ni(cycP) | DT, the modelled coverage consisted in one cocatalyst and two dithiol molecules adsorbed in a $p(3\times2)$ cell.

Modelling of the adsorption modes in ZnSe | Ni(cycP) | DT

In ZnSe | Ni(cycP) | EDT, the monodentate adsorption of EDT was modelled on site **H** (Figure 6C) as this is the only position from which this capping ligand can interact with the CO_2RR intermediates adsorbed on the Ni(cycP) cocatalyst. To model the bidentate mode of EDT, the sites **H-B** were considered as the ligand in this position has a minimum distance of 3.31 Å to the Ni(cycP) cocatalyst and its binding energy was expected to be very similar to that on the other available Zn sites further away from the cocatalyst (Table S5). This was verified by adsorbing EDT on the sites **D-E**, which resulted in a binding energy for EDT that differs only by 0.01 eV from that on **H**-**B**.

For HexDT and OctDT, due to the flexibility of the ligand chains, it was possible to identify more than one bidentate adsorption besides all the monodentate ones. Consequently, for all the positions from **A** to **G** (Figure 6C), the dithiol was directed once along the *a* and *b* axes, and diagonally, excluding orientations that would bring the dithiols on the Zn sites immediately around the Ni(cycP), as they were deemed unlikely due to steric hindrance.

Neglection of the bidentate ligand in ZnSe | Ni(cycP) | DT

In ZnSe | Ni(cycP) | DT, of the two dithiols in the $p(3\times2)$ cell, one was initially modelled as bidentate since this was found to be the most favorable adsorption in the system ZnSe | DT, while the other one was considered to be monodentate to interact with the CO_2RR intermediates adsorbed on the cocatalyst. In subsequent reactivity studies, however, the bidentate ligand was removed from the calculation for two main reasons. Firstly, this ligand does not interact with the second coordination sphere of the cocatalyst, and therefore, it may not influence the energetics of the CO_2RR intermediates; and secondly, the distance between the neighboring Zn sites (5.75 Å and 4.06 Å along the *a* and *b* axes, respectively), is long enough to minimize the interaction between the adsorbed dithiols. Accordingly, the binding energy of two dithiols in the $p(3\times2)$ cell is almost equal to the sum of the individual binding energies, as shown in Table S6 for a sample of control combinations computed for HexDT. In short, an HexDT ligand in the most stable bidentate mode was modelled along with another HexDT molecule in the available monodentate configurations. Based on these results, it is reasonable to assume that this approach will also be valid for EDT and OctDT as these ligands differ from HexDT only in the chain length, but the orientations of the adsorption are the same.

Supporting Tables

Table S1. Optimisation of photocatalytic CO_2 reduction using ZnSe-BF_4 and dithiols. Unless otherwise stated, conditions were: 0.5 µM QD, 0.1 M AA/$NaHCO_3$, 3 mL under CO_2 flow (4 sccm); 100 mW cm^{-2}, AM 1.5G, λ > 400 nm, 10 h irradiation, 25 °C.

Catalyst	Ligand loading / µM	Co-catalyst loading / µM	Dithiol length / Å	$n(H_2) \pm \sigma$ / µmol	$n(CO) \pm \sigma$ / µmol	CO selectivity[a]
**ZnSe | dithiol (pH 6.5)**						
ZnSe	0	0	0	16.7 ± 4.60	0.15 ± 0.02	0.9% ± 0.1%
ZnSe | EDT	50	0	4.3	5.29 ± 0.79	0.95 ± 0.19	15.1% ± 2.6%
ZnSe | BuDT	50	0	6.8	5.84 ± 0.99	0.46 ± 0.06	7.4% ± 0.4%
ZnSe | HexDT	50	0	9.3	8.18 ± 1.23	0.14 ± 0.03	1.7% ± 0.3%
ZnSe | OctDT	50	0	11.7	4.21 ± 0.63	0.27 ± 0.04	5.9% ± 0.5%
ZnSe | BenzDT	50	0	6.4	7.49 ± 0.97	0.19 ± 0.01	2.6% ± 0.3%
ZnSe | HO-EtSH	50	0	-	14.5 ± 7.89	0.56 ± 0.06	4.5% ± 2.5%
ZnSe | BuSH	50	0	-	16.8 ± 6.38	0.17 ± 0.04	1.1% ± 0.6%
**ZnSe | NiCycP | dithiol (pH 5.5)**						
ZnSe | NiCycP	0	10	0	25.7 ± 3.69	1.09 ± 0.18	4.0% ± 0.2%
ZnSe | NiCycP | EDT	25	10	4.3	17.6 ± 2.64	1.21 ± 0.18	6.4% ± 0.3%
ZnSe | NiCycP | BuDT	25	10	6.8	11.6 ± 1.74	3.78 ± 0.46	24.5% ± 2.0%
ZnSe | NiCycP | HexDT	25	10	9.3	6.19 ± 0.93	4.05 ± 0.25	39.6% ± 1.9%
ZnSe | NiCycP | OctDT	25	10	11.7	5.82 ± 0.87	2.97 ± 0.24	33.8% ± 1.8%
ZnSe | NiCycP | BenzDT	25	10	6.4	11.7 ± 0.59	1.45 ± 0.07	10.9% ± 0.5%
ZnSe | NiCycP | HO-HexSH	25	10	-	22.4 ± 1.51	3.10 ± 0.16	12.2% ± 0.6%
ZnSe | NiCycP | HexSH	25	10	-	17.9 ± 0.89	1.15 ± 0.06	6.0% ± 0.3%

Table S2. Control experiments for the photocatalytic CO_2 reduction using dithiol functionalised ZnSe-BF_4 QDs. Unless otherwise stated, conditions were: 0.5 µM ZnSe-BF_4, 0.1 M AA/$NaHCO_3$ pH 6.5 (w/o cocatalyst) or 0.1 M AA pH 5.5 (w/ co-catalyst), 3 mL water under CO_2 flow (4 sccm); 100 mW cm^{-2}, AM 1.5G, λ > 400 nm, 25°C.

description	time / h	$n(CO) \pm \sigma$ / µmol	$n(H_2) \pm \sigma$ / µmol
standard experiment (ZnSe | EDT)	10	0.95 ± 0.19	5.29 ± 0.79
no EDT	10	0.15 ± 0.02	16.7 ± 4.60
no AA	20	not detected	not detected
no ZnSe QDs	20	not detected	not detected
no light	10	not detected	not detected
Standard experiment (ZnSe | NiCycP | HexDT)	10	4.05 ± 0.25	6.19 ± 0.93
no NiCycP (ZnSe | HexDT)	10	0.75 ± 0.08	12.07 ± 0.60
no HexDT	10	1.09 ± 0.18	25.7 ± 3.69
no AA	20	not detected	not detected
no ZnSe QDs	20	not detected	not detected
no light	10	not detected	not detected

Table S3. pH controls. Unless otherwise stated, standard conditions were: 0.5 μM QD, 0.1 M AA/$NaHCO_3$, 3 mL water under CO_2 flow (4 sccm); 100 mW cm^{-2}, AM 1.5G, λ > 400 nm, 10 h irradiation, 25°C.

Catalyst	Ligand loading / μM	Co-catalyst loading / μM	pH	n(H_2) ± σ / μmol	n(CO) ± σ / μmol	CO selectivity[a]
**ZnSe | dithiol**						
ZnSe | EDT	50	0	6.5	5.29 ± 0.79	0.95 ± 0.19	15.1% ± 2.6%
ZnSe | EDT	50	0	5.5	17.2 ± 0.86	0.68 ± 0.08	0.9%
ZnSe | HexDT	50	0	6.5	8.18 ± 1.23	0.14 ± 0.03	1.7% ± 0.3%
ZnSe | HexDT	50	0	5.5	8.43 ± 0.42	0.11 ± 0.01	1.3%
**ZnSe | NiCycP | dithiol**						
ZnSe | NiCycP | EDT	25	10	6.5	9.54 ± 1.05	0.42 ± 0.02	4.2% ± 0.6%
ZnSe | NiCycP | EDT	25	10	5.5	17.6 ± 2.64	1.21 ± 0.18	6.4% ± 0.3%
ZnSe | NiCycP | HexDT	25	10	6.5	7.25 ± 0.36	0.97 ± 0.22	11.8% ± 2.5%
ZnSe | NiCycP | HexDT	25	10	5.5	6.19 ± 0.93	4.05 ± 0.25	39.6% ± 1.9%

Table S4. Gibbs binding energies (in eV), ΔG, for a single capping ligand in a mono- and bidentate configuration in ZnSe | DT.

System	Monodentate	Bidentate
ZnSe | EDT	-0.12	-0.27
ZnSe | HexDT	-0.56	-0.98
ZnSe | OctDT	-0.55	-0.72

Table S5. Calculated ΔG values (in eV) for a single capping ligand in a mono- and bidentate configuration in the *p*(3×2) supercell ZnSe | Ni(cycP) | DT.

System	Site	ΔG
**Monodentate adsorption**		
ZnSe | Ni(cycP) | EDT	**H**	-0.04
ZnSe | Ni(cycP) | HexDT	**A**	-0.64
ZnSe | Ni(cycP) | HexDT	**B**	-0.66
ZnSe | Ni(cycP) | HexDT	**C**	-0.52
ZnSe | Ni(cycP) | HexDT	**D**	-0.49
ZnSe | Ni(cycP) | HexDT	**E**	-0.46
ZnSe | Ni(cycP) | HexDT	**F**	-0.37
ZnSe | Ni(cycP) | HexDT	**G**	-0.53
ZnSe | Ni(cycP) | OctDT	**A**	-0.91
ZnSe | Ni(cycP) | OctDT	**B**	-0. 90
ZnSe | Ni(cycP) | OctDT	**C**	-0.64
ZnSe | Ni(cycP) | OctDT	**D**	-0.56
ZnSe | Ni(cycP) | OctDT	**E**	-0.87
ZnSe | Ni(cycP) | OctDT	**F**	-0.68
ZnSe | Ni(cycP) | OctDT	**G**	-0.79
**Bidentate adsorption**		
ZnSe | Ni(cycP) | EDT	**H-B**	-0.29
ZnSe | Ni(cycP) | HexDT	**A-B**	-0.83
ZnSe | Ni(cycP) | HexDT	**A-H**	-0.64
ZnSe | Ni(cycP) | HexDT	**B-D**	-0.77
ZnSe | Ni(cycP) | HexDT	**B-E**	-0.86
ZnSe | Ni(cycP) | HexDT	**C-D**	-0.64
ZnSe | Ni(cycP) | HexDT	**C-G**	-0.73
ZnSe | Ni(cycP) | HexDT	**C-H**	-0.96
ZnSe | Ni(cycP) | HexDT	**D-F**	-1.11
ZnSe | Ni(cycP) | HexDT	**E-G**	-1.15
ZnSe | Ni(cycP) | HexDT	**E-H**	-0.77
ZnSe | Ni(cycP) | HexDT	**F-H**	-0.87
ZnSe | Ni(cycP) | OctDT	**A-B**	-0.79
ZnSe | Ni(cycP) | OctDT	**A-D**	-0.78
ZnSe | Ni(cycP) | OctDT	**B-F**	-0.92
ZnSe | Ni(cycP) | OctDT	**C-E**	-0.93
ZnSe | Ni(cycP) | OctDT	**F-G**	-1.44
ZnSe | Ni(cycP) | OctDT	**G-H**	-1.05

Table S6. Calculated ΔG values (in eV) for two HexDT ligands adsorbed in the *p*(3×2) supercell ZnSe | Ni(cycP) | HexDT benchmarked against the sum of the individual binding energies in the same cell.

Site	ΔG combined system	Sum of ΔGs
**E-G + A**	-1.73	-1.79
**E-G + B**	-1.82	-1.81
**E-G + C**	-1.64	-1.67
**E-G + D**	-1.67	-1.64

Table S7. Relative Gibbs energies (in eV) of the CO_2RR intermediates, ΔG_i, in ZnSe | Ni(cycP) and ZnSe | Ni(cycP) | DT. Energies are referenced to ZnSe | Ni(cycP) or ZnSe | Ni(cycP) | DT, and the CO_2 and H_2 molecules in the gas phase.

System	Site	ΔG_{*CO2}	ΔG_{*COOH}	ΔG_{*CO}
ZnSe | Ni(cycP)	**-**	-0.26	0.25	-0.06
ZnSe | Ni(cycP) | EDT	**H**	-0.35	0.22	-0.10
ZnSe | Ni(cycP) | HexDT	**A**	0.05	0.41	0.14
ZnSe | Ni(cycP) | HexDT	**B**	-0.04	0.32	0.20
ZnSe | Ni(cycP) | HexDT	**C**	-0.36	0.48	-0.06
ZnSe | Ni(cycP) | HexDT	**D**	-0.24	0.33	0.05
ZnSe | Ni(cycP) | HexDT	**E**	-0.35	0.21	-0.04
ZnSe | Ni(cycP) | HexDT	**F**	-0.37	0.20	-0.03
ZnSe | Ni(cycP) | HexDT	**G**	-0.28	0.27	0.01
ZnSe | Ni(cycP) | OctDT	**A**	0.01	0.87	0.31
ZnSe | Ni(cycP) | OctDT	**B**	0.01	0.59	0.30
ZnSe | Ni(cycP) | OctDT	**C**	-0.30	0.60	-0.01
ZnSe | Ni(cycP) | OctDT	**D**	-0.27	0.28	0.01
ZnSe | Ni(cycP) | OctDT	**E**	0.21	0.75	0.50
ZnSe | Ni(cycP) | OctDT	**F**	-0.04	0.50	0.27
ZnSe | Ni(cycP) | OctDT	**G**	-0.02	0.79	0.22

Supporting Figures

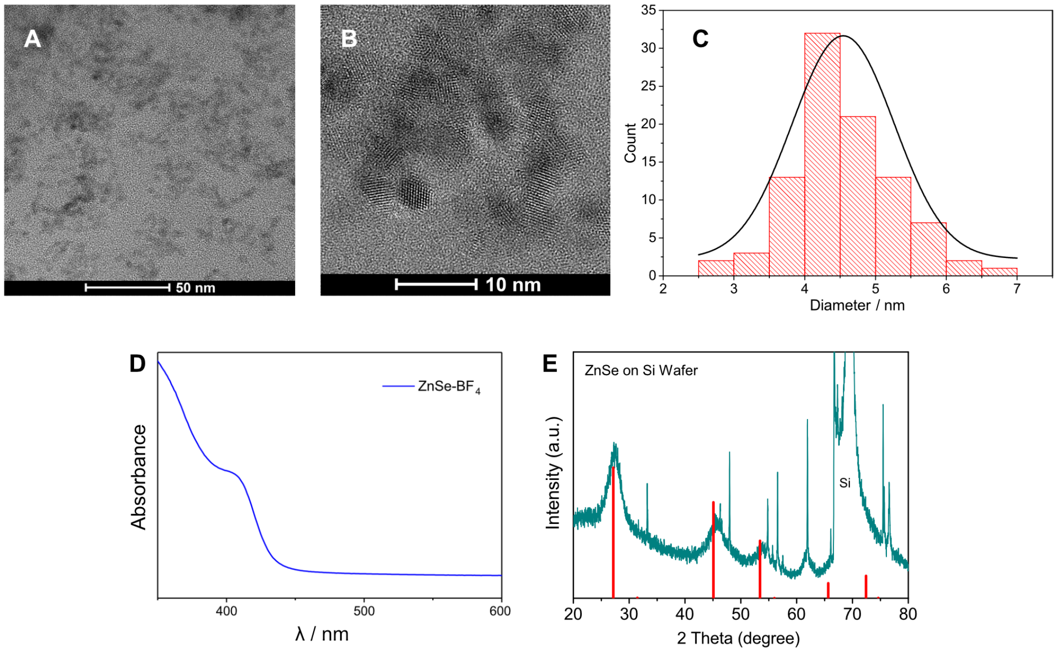

Figure S1. Characterization of ZnSe-BF_4 QDs. (A-B) Transmission electron micrographs; (C) Particle size distribution determined by TEM; (D) UV-vis absorption spectrum, 10 µL ZnSe-BF_4 stock solution in 1 mL water, 1 cm path length, room temperature; (E) Powder X-ray diffractogram of ZnSe-BF_4 on Si wafer (green) overlaid with cubic zinc blende ZnSe reference (PDF 01-071-5978, red), the unassigned signals originate from the Si wafer.

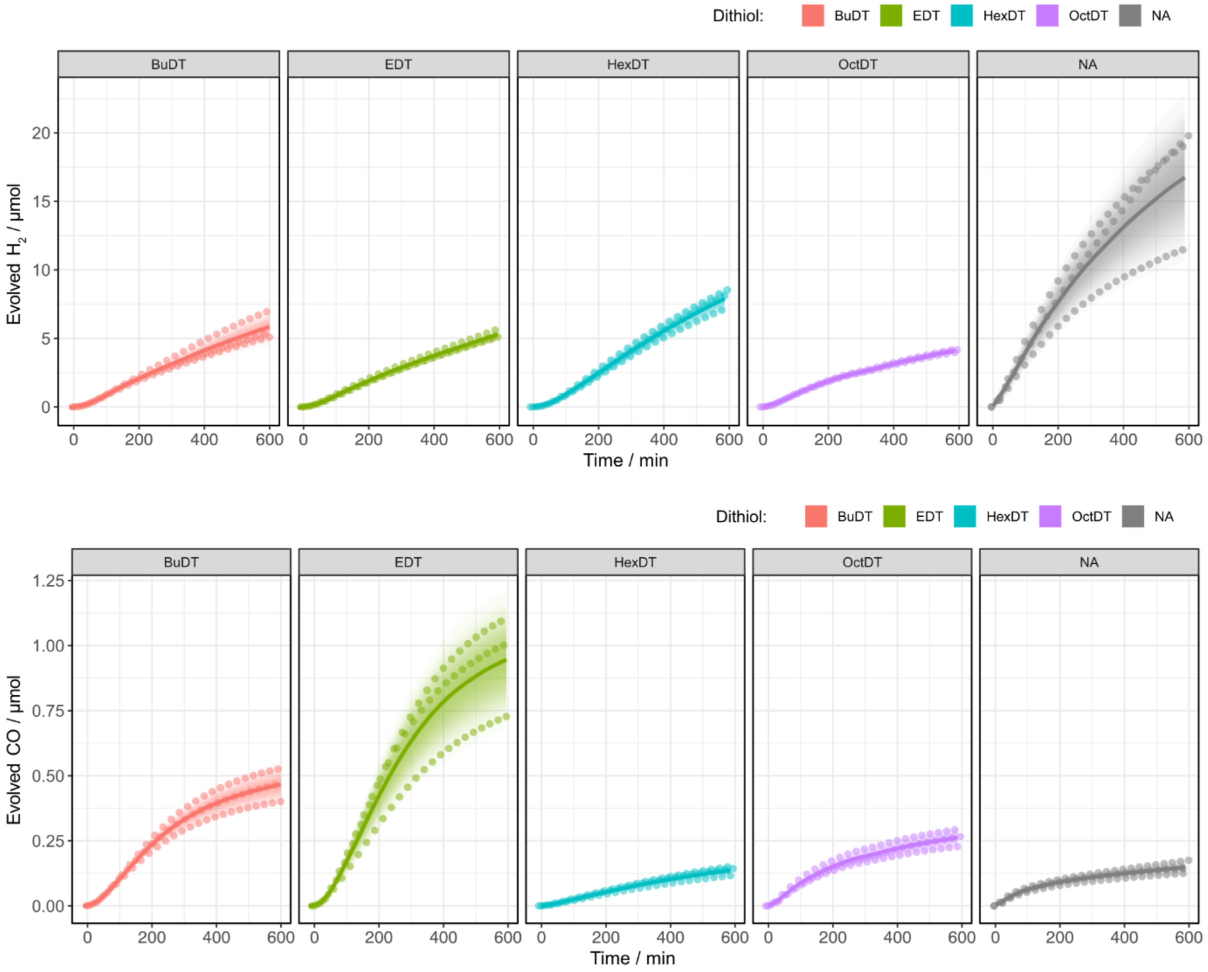

Figure S2. Photocatalytic CO_2 reduction in the presence of ZnSe | dithiol: Conditions: 0.5 µM ZnSe-BF_4, 50 µM dithiol; 0.1 M AA/$NaHCO_3$, pH 6.5, 3 mL under CO_2 flow (4 sccm); 100 mW cm^{-2}, AM 1.5G, λ > 400 nm, 25 °C.

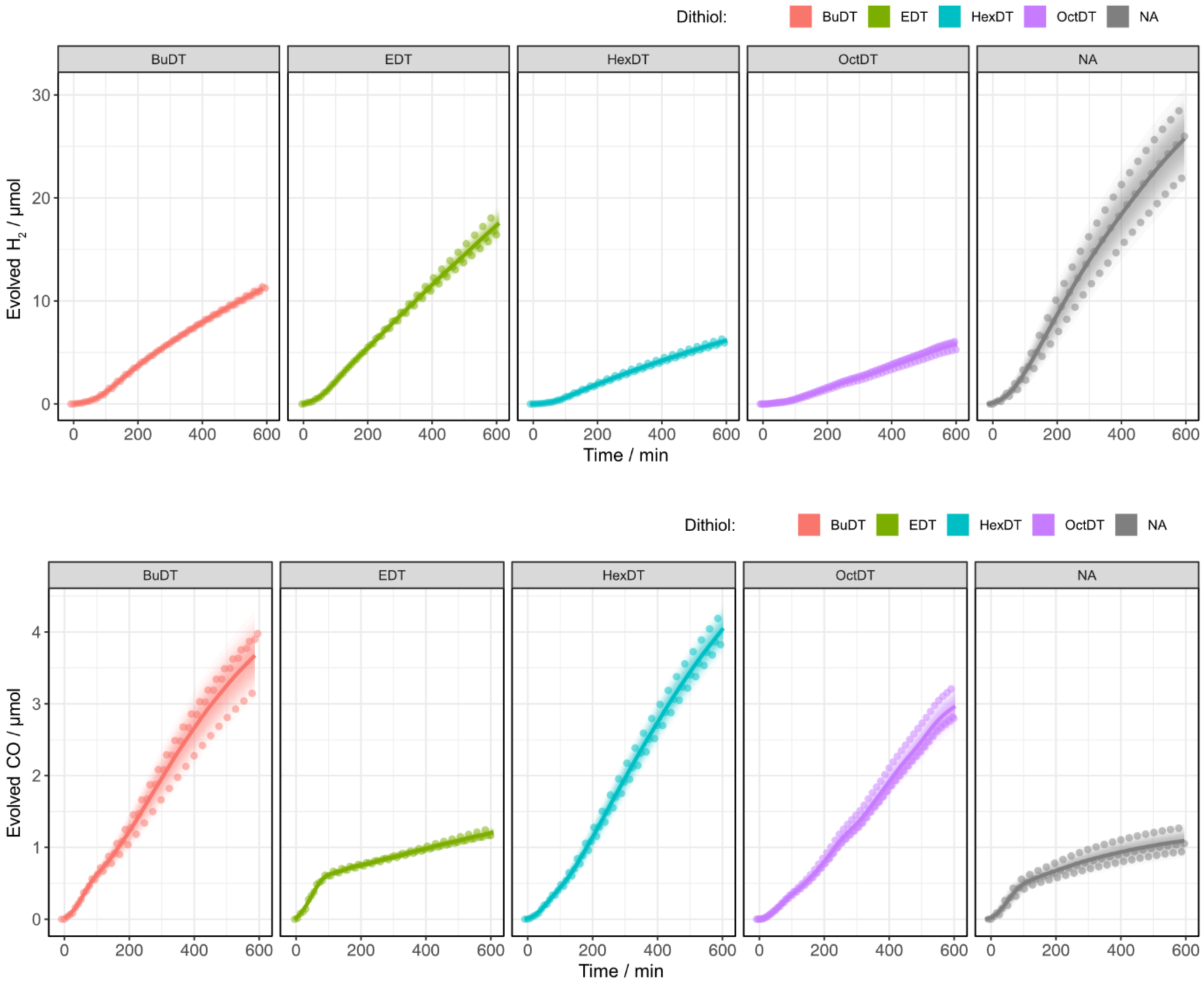

Figure S3. Photocatalytic CO_2 reduction in the presence of ZnSe | Ni(cycP) | dithiol. Conditions: 0.5 µM ZnSe-BF_4, 25 µM dithiol, 10 µM Ni(cycP), 0.1 M AA, pH 5.5, 3 mL under CO_2 flow (4 sccm); 100 mW cm^{-2}, AM 1.5G, λ > 400 nm, 25°C.

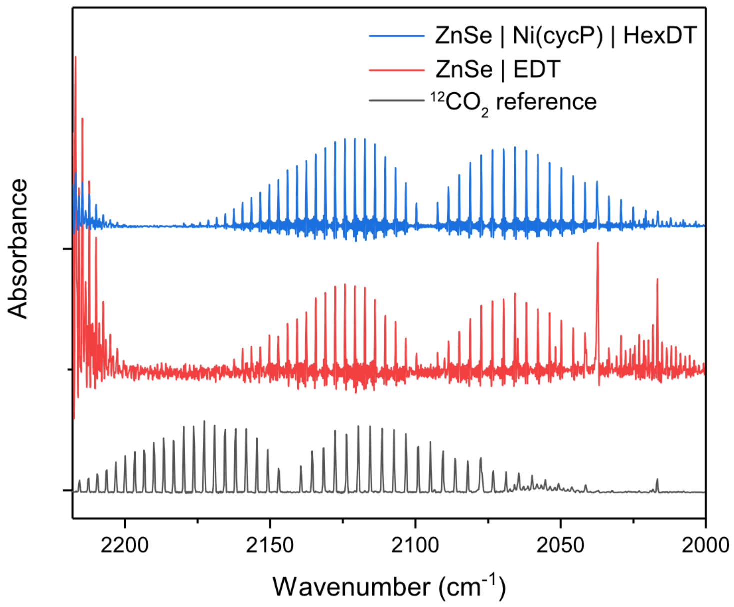

Figure S4. Isotopic labelling: Gas-phase transmission infrared (IR) spectra of the CO vibration depending on the employed CO_2 isotopologue. Samples ZnSe | EDT and ZnSe | Ni(CycP) | HexDT and under an atmosphere of $^{13}CO_2$ compared to a reference spectrum of ^{12}CO. Conditions: AM 1.5G, λ > 400 nm, 100 mW cm^{-2}, 0.5 µM ZnSe-BF$_4$, 0.1 M AA/NaHCO$_3$ pH 6.5 (w/o Ni(cycP), AA pH 5.5 (w/ Ni(cycP), CO_2, 16 h irradiation. Products accumulated in the headspace.

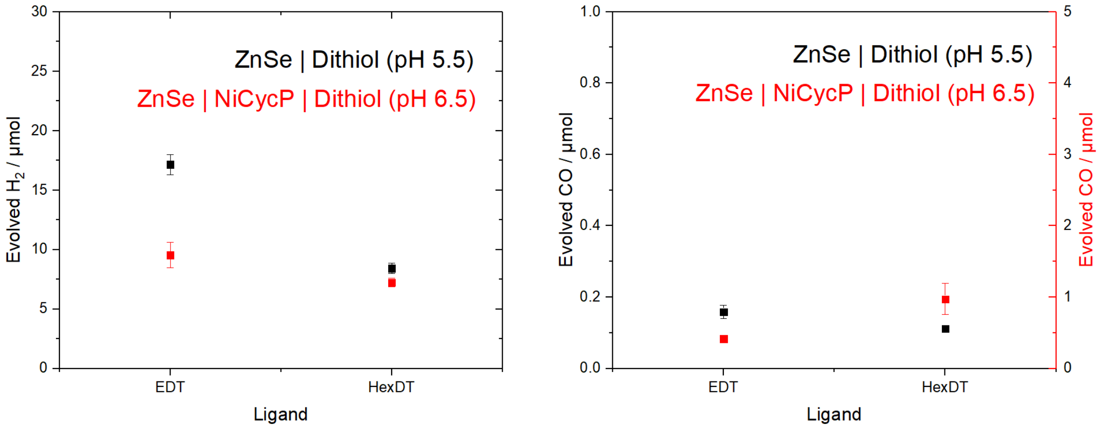

Figure S5. pH control Photocatalytic CO_2 reduction in the presence of ZnSe | dithiol: (Reverse) pH controls. Conditions: 0.5 µM ZnSe-BF4, 0.1 M AA/NaHCO$_3$, for ZnSe | dithiol: 50 µM dithiol, for ZnSe | Ni(cycP) | dithiol: 25 µM dithiol, 10 µM Ni(cycP); 3 mL solution under CO2 flow (4 sccm); 100 mW cm^{-2}, AM 1.5G, λ > 400 nm, 10 h irradiation, 25 °C. Based on two independent replicates.

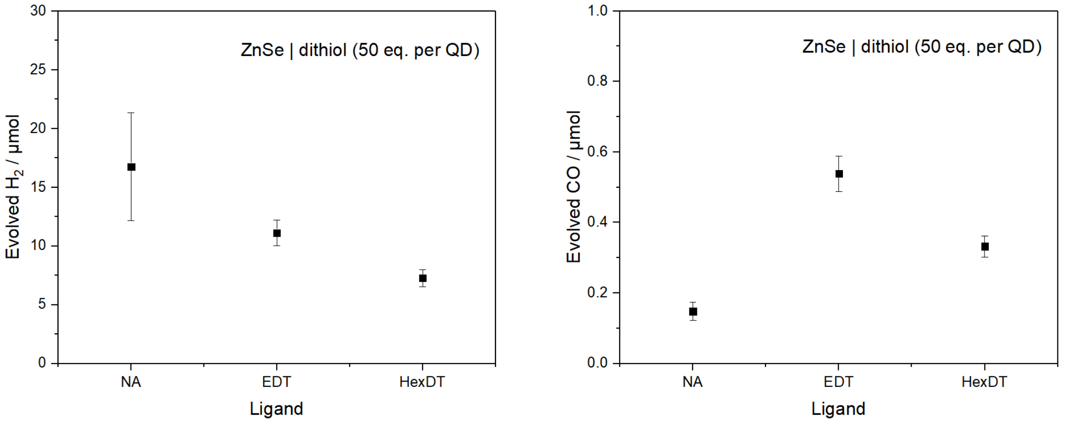

Figure S6. Ligand loading control. Photocatalytic CO2 reduction in the presence of ZnSe | dithiol: loading control. Conditions: 0.5 µM QD, 25 µM dithiol, 0.1 M AA/$NaHCO_3$, pH 6.5, 3 mL under CO_2 flow (4 sccm); 100 mW cm^{-2}, AM 1.5G, λ > 400 nm, 25 °C. Based on two independent replicates.

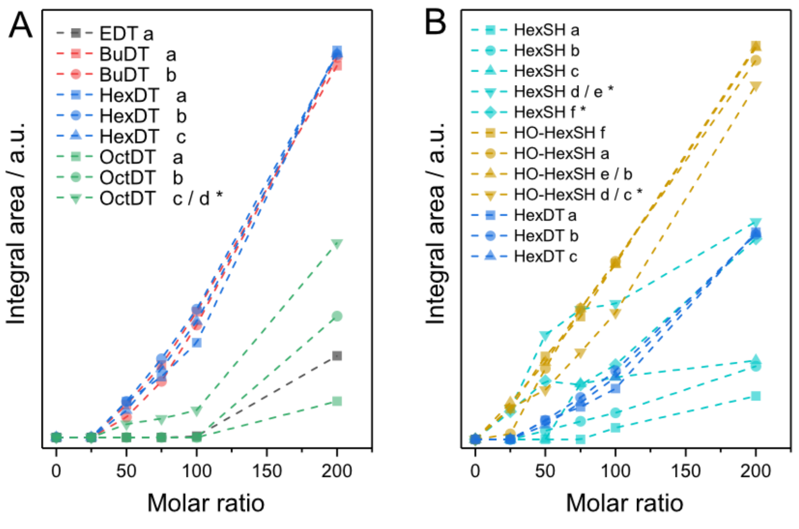

Figure S7. Integrated area of the signals from ^{1}H-NMR titration of dithiol analogues to ZnSe QDs. Signals normalised to one proton. (A) Various dithiols and (B) HexDT in comparison to dithiol analogues with 6 carbon centres.

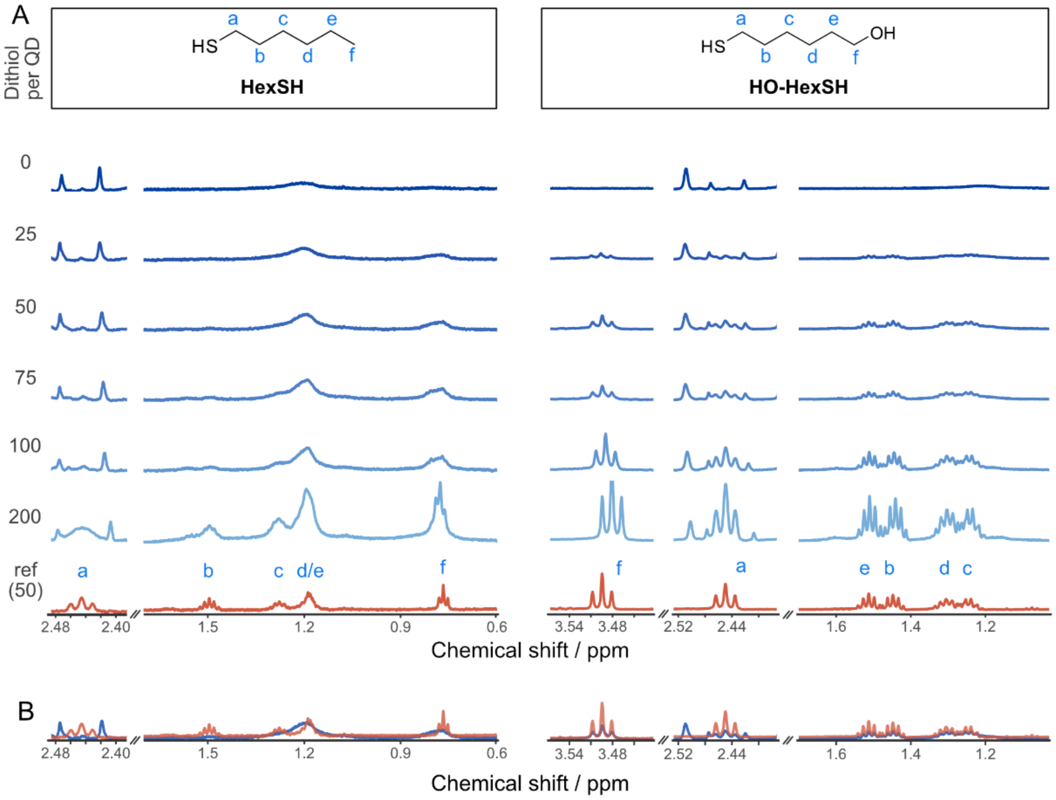

Figure S8. Interactions of dithiol-analogues (1,6-mercaptohexanol (HO-HexSH) and 1-hexanethiol (HexSH)) with ZnSe-QDs. (A) 1H-NMR spectroscopy titration experiment with aliquots of ligand (in ACN-d3) being added to a D_2O suspension of 2 µM ZnSe-BF4 QDs. Intensities are not to scale (in-between ligands) and were adjusted for optimal visibility. (B) Overlay of the reference ligand spectrum (100 µM) in the absence (orange) and presence of ZnSe (2 µM, blue).

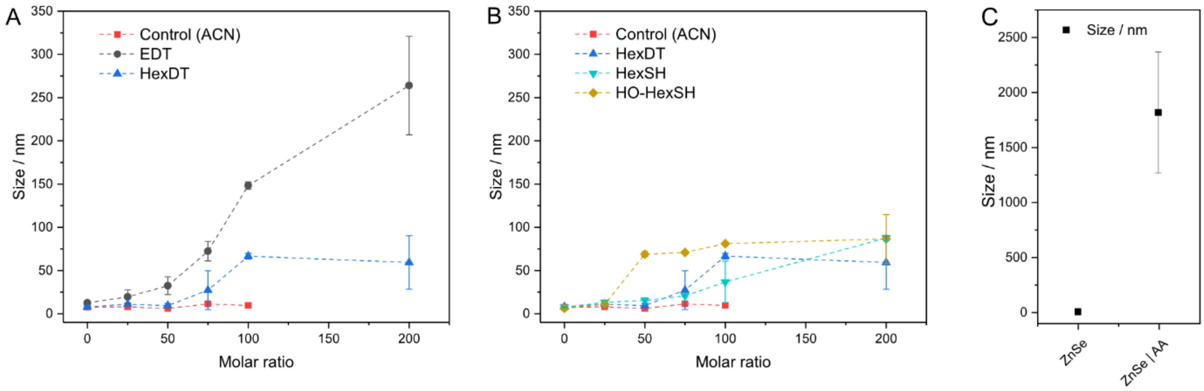

Figure S9. Dynamic light scattering (DLS) data of ZnSe-BF_4 QDs (0.5 µM) and in the presence of dithiol ligands (A) and analogues (B) in aqueous solution (neutral pH). Shown is the number mean. Dithiol ligands were added stepwise from a stock solution (0.5 mM in acetonitrile). The control titration consists of only solvent (acetonitrile) added to a suspension of QDs to exclude solvent and time effects. Shown is the number mean. The lines were added to guide the eye. (C) DLS measurements of ZnSe-BF_4 QDs in water (neutral pH), in the presence of AA (0.1 M, pH 5.5), number mean.

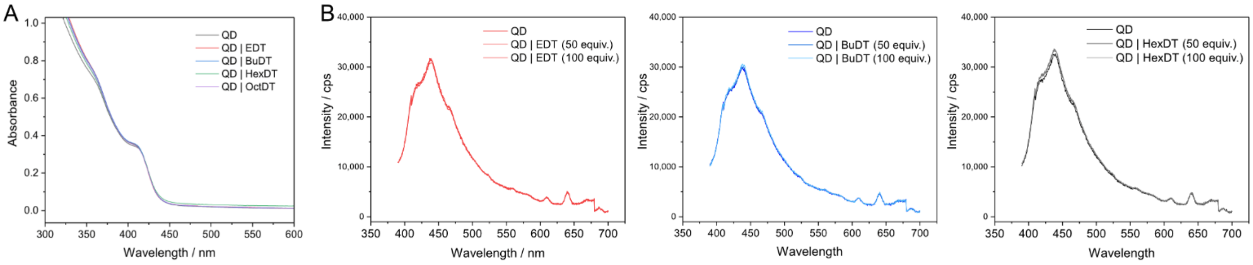

Figure S10. Photophysical characterization of the influence of dithiols on ZnSe QDs. (A) UV-vis absorption spectroscopy. (B) Photoluminesence spectroscopy (emission intensity) of various ZnSe QDs in the presence and absence of various dithiols (λ_{ex} = 360 nm).

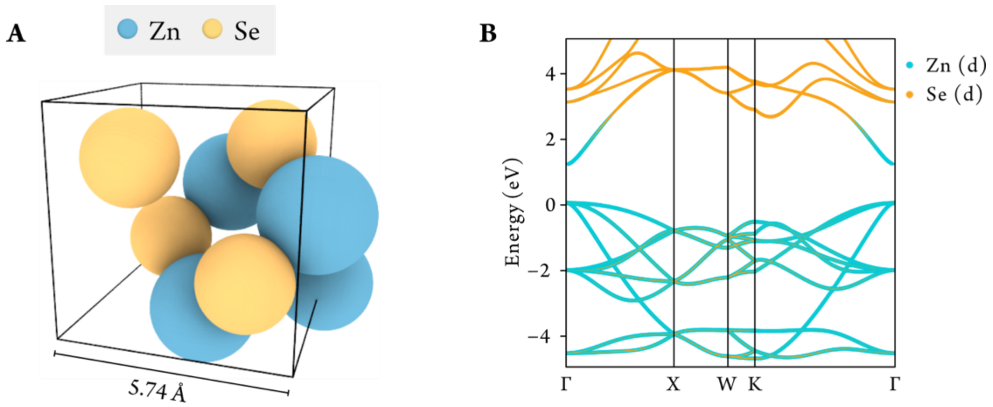

Figure S11. A) Representation of the bulk structure of the ZnSe used for the construction of the ZnSe QD surface. B) Band structure of the ZnSe bulk represented in A), calculated at the DFT-PBE level (see Computational Methods for details).

Supporting References

[1] Neri G.; Forster, M., Walsh, J. J.; Robertson, C. M.; Whittles, T. J.; Farràs, P.; and Cowan, A. J.; *Chem. Commun.*, **2016**, *52*, 14200–14203.

[2] Sahm, C. D.; Mates-Torres, E.; Eliasson, N.; Sokołowski K.; Wagner, A.; Dalle, K. E.; Huang, Z.; Scherman, O. A.; Hammarström, L.; García-Melchor, M.; and Reisner, E. *Chem. Sci.*, **2021**, *12*, 9078–9087.

[3] R Core Team. (2020). R: A language and environment for statistical computing. Vienna, Austria: R Foundation for Statistical Computing. Retrieved from https://www.R-project.org/

[4] Wickham et al., Welcome to the Tidyverse. *Journal of Open Source Software*. **2019** *4*, 1686, (https://doi.org/10.21105/joss.01686)

[5] Pav, S.-E., Documentation for Geom_cloud function. Retrieved from https://www.rdocumentation.org/packages/ggallin/versions/0.1.1/topics/geom_cloud (**2020**)

[6] Perdew, J. P.; Burke, K.; Ernzerhof, M. *Physical Review Letters,* **1996**, *77*, 3865–3868

[7] Grimme, S., Ehrlich, S. & Goerigk, L. *J. Comput. Chem.* **2011**, 32, 1456–1465

[8] Kresse, G.; Furthmüller, J. *Physical Review B*, **1996**, *54*, 11169–11186

[9] Blöchl, P. E. *Physical Review B*, **1994**, *50*, 17953–17979

[10] Jain, A.; Ong, S. P.; Hautier, G.; Chen, W.; Richards, W. D.; Dacek, S.; Cholia, S.; Gunter, D.; Skinner, D.; Ceder, G. *et al. APL Materials* **2013**, *1*, 011002.

[11] Zhao, C.-Z; Huang, Y.; Qi, X.-L.; Wang, Y.-L. *Appl. Phys. A*, **2022**, *128*, 32.

[12] Larsen et al, *J. Phys. Condens. Matter,* 2017, **29**, 273002.

[13] Chai, J.-D.; Head-Gordon, M. *Phys. Chem. Chem. Phys.* **2008**, *10*, 6615–6620.

[14] Gaussian 09, Revision **E.01**, M. J. Frisch, G. W. Trucks, H. B. Schlegel, G. E. Scuseria, M. A. Robb, J. R. Cheeseman, G. Scalmani, V. Barone, G. A. Petersson, H. Nakatsuji, X. Li, M. Caricato, A. Marenich, J. Bloino, B. G. Janesko, R. Gomperts, B. Mennucci, H. P. Hratchian, J. V. Ortiz, A. F. Izmaylov, J. L. Sonnenberg, D. Williams-Young, F. Ding, F. Lipparini, F. Egidi, J. Goings, B. Peng, A. Petrone, T. Henderson, D. Ranasinghe, V. G. Zakrzewski, J. Gao, N. Rega, G. Zheng, W. Liang, M. Hada, M. Ehara, K. Toyota, R. Fukuda, J. Hasegawa, M. Ishida, T. Nakajima, Y. Honda, O. Kitao, H. Nakai, T. Vreven, K. Throssell, J. A. Montgomery, Jr., J. E. Peralta, F. Ogliaro, M. Bearpark, J. J. Heyd, E. Brothers, K. N. Kudin, V. N. Staroverov, T. Keith, R. Kobayashi, J. Normand, K. Raghavachari, A. Rendell, J. C. Burant, S. S. Iyengar, J. Tomasi, M. Cossi, J. M. Millam, M. Klene, C. Adamo, R. Cammi, J. W. Ochterski, R. L. Martin, K. Morokuma, O. Farkas, J. B. Foresman, and D. J. Fox, Gaussian, Inc., Wallingford CT, **2009**.x

[15] Ehlers, A. W.; Böhme, M.; Dapprich, S.; Gobbi, A.; Höllwarth, A.; Jonas, V.; Köhler, K. F.; Stegmann, R.; Veldkamp, A.; Frenking, G. *Chem. Phys. Lett.* **1993**, *208*, 111–114.

[16] E. R. Johnson, S. Keinan, P. Mori-Sánchez, J. Contreras-García, A. J. Cohen and W. Yang, *J. Am. Chem. Soc.*, 2010, **132**, 6498–6506.

[17] Otero-de-la-Roza, A.; Johnson, E. R.; Luaña, V. *Comput. Phys. Commun*. **2014**, *185*, 1007−1018

End of Supporting Information
